# Accurate density functional theory for noncovalent interactions in charged systems

**DOI:** 10.1126/sciadv.adz8521

**Published:** 2026-04-22

**Authors:** Heng Zhao, Balázs D. Lőrincz, Tobias Henkes, Dénes Berta, Péter R. Nagy, Alexandre Tkatchenko, Stefan Vuckovic

**Affiliations:** ^1^ Department of Chemistry, University of Fribourg, Fribourg 1700, Switzerland.; ^2^ Department of Physical Chemistry and Materials Science, Faculty of Chemical Technology and Biotechnology, Budapest University of Technology and Economics, Budapest H-1111, Hungary.; ^3^ HUN-REN–BME Quantum Chemistry Research Group, Műegyetem rkp. 3, H-1111 Budapest, Hungary.; ^4^ MTA–BME Lendület Quantum Chemistry Research Group, Műegyetem rkp. 3, H-1111 Budapest, Hungary.; ^5^ Department of Physics and Materials Science, University of Luxembourg, Luxembourg City L-1511, Luxembourg.

## Abstract

Accurately modeling noncovalent interactions (NCIs) involving charged systems remains an outstanding challenge in density functional theory (DFT), with implications across natural and life sciences, engineering, e.g., in biochemistry, catalysis, and materials science. For these interactions, the interplay between electrostatics, polarization, and dispersion leads to systematic errors of up to tens of kilocalories per mole in standard dispersion–enhanced DFT methods. We solve this problem by introducing (r^2^SCAN+MBD)@HF, a DFT method without empirically fitted parameters that combines the r^2^SCAN functional and many-body dispersion, both evaluated on Hartree-Fock densities. We show that the unique synergy of these three components enables balanced treatment of short- and long-range correlation, which is crucial for accurate description of NCIs involving charged systems. Evaluations on standard benchmarks show that (r^2^SCAN+MBD)@HF significantly improves accuracy for NCIs involving charged systems while maintaining robust performance for neutral systems. Further tests on the Metal Ion Protein Clusters dataset introduced here demonstrate its improved accuracy for metal-protein interactions, including cases where the metal ion is surrounded by negatively charged ligands for which standard DFT can completely break down. Given the ubiquity of such interactions, (r^2^SCAN+MBD)@HF is broadly applicable from biochemistry and materials science, including for generating high-quality data to train machine-learning force fields.

## Teaser

Nonempirical DFT resolves accurate simulation of charged noncovalent interactions.

## INTRODUCTION

Noncovalent interactions (NCIs) involving charged systems (charged NCIs for simplicity) are ubiquitous across scientific disciplines, such as in acid-base chemistry, electrochemistry, redox chemistry, coordination chemistry, ionic crystals and liquids, ionization and electron attachment processes, and charge conduction and transfer. For example, in biology, charged NCIs stabilize enzymatic transition states (*1*), mediate protein folding via salt bridges between charged amino acid side chains (*2*), and regulate ion transport (*3*). In catalysis, ionic complex stability dictates reaction pathways and selectivity [e.g., in Brønsted and Lewis acid catalysis; (*4*)]. In materials science, they enable gas separation in metal-organic frameworks (MOFs) (*5*) and play a crucial role in the design of batteries by governing alkali metal intercalation in electrode materials (*6*). Thus, accurate computational models of charged NCIs are essential, spanning applications from ionic liquids (*7*) to electrochemical processes in energy storage systems (*8*).

When it comes to computational simulations of NCIs, including those involving charged species, benchmark-level accuracy is typically achieved using high-level wave function–based methods, such as coupled cluster with single, double, and perturbative triple excitations [CCSD(T)] (*9*) and quantum diffusion Monte Carlo (*10*). Despite advances in cost reduction (*11*–*14*), these wave function methods remain impractical for biological or condensed-phase systems exceeding hundreds of atoms.

The unparalleled trade-off between accuracy and cost has made density functional theory (DFT) the workhorse for NCI simulations (*15*–*18*). While DFT initially struggled with dispersion interactions, this issue has been largely mitigated, especially for neutral cases, by various dispersion methods (*19*–*27*). However, the growing interest in charged NCI datasets (*28*–*30*), including the extensive DES15K benchmark set (*31*), has revealed the major limitations of dispersion-enhanced DFT for these interactions (*17*). Namely, while dispersion-enhanced DFT typically performs well for neutral complexes, with interaction energy errors around 0.5 kcal/mol, its errors for charged species can be up to 10 times larger, regardless of the chosen method [“D3” (*22*, *23*), “D4” (*32*, *33*), “XDM” (*19*, *20*), “TS” (*21*), or “MBD” (*26*, *27*)]. This issue is systematic, as recently demonstrated by Johnson’s study of ∼15,000 (*31*) NCI complexes (*17*).

Computational simulations of charged NCIs fundamentally differ from neutral ones, especially for interactions between metallic cations and neutral molecules. A prototypical example is the Li^+^-benzene complex (*17*), where the strong inhomogeneous electric field from Li^+^’s localized positive charge distorts the benzene electron cloud, inducing partial electron transfer. From the perspective of the theory of intermolecular interactions, describing such interactions in inhomogeneous electric fields is notoriously challenging as electrostatics, polarization, and dispersion become inherently coupled (*34*). This coupling critically influences a broad range of (bio)chemical processes, such as selectivity in biological ion channels [see (*3*)]. Further complexity arises because external charges substantially alter dispersion interactions, stabilizing or destabilizing molecular binding depending on the charge sign (*34*).

Within DFT, accurately describing strongly polarized complexes under electric fields is particularly challenging due to delocalization errors, often (*35*, *36*) termed “the greatest outstanding challenge in DFT” (*35*). Another critical issue is accurately capturing electrostatics, polarization, and dispersion and their coupling, requiring careful balancing of short- and long-range correlation without double-counting effects (*37*, *38*). Along these lines, dispersion methods in DFT compensate for the long-range correlation missed by (semilocal) DFT approximations (*15*–*17*). However, dispersion methods must be balanced with base DFT to avoid double-counting of correlation effects [see (*37*, *38*)]. Although general, this issue is particularly severe for charged NCIs, due to the interplay of polarization, density errors, and poor transferability of dispersion methods trained on neutral NCIs. Simply put, DFT typically underbinds neutral NCIs, so dispersion methods improve their interaction energies. In contrast, for charged NCIs, where DFT already overbinds, adding an attractive dispersion energy only worsens the overbinding. Thus, developing a dispersion-enhanced DFT approximation that describes both neutral and charged NCIs remains a major challenge.

Resolving this challenge with charged NCIs is crucial, especially today as machine-learning force fields (MLFFs) trained on DFT data expand the reach of DFT quality modeling to larger systems and longer dynamics simulations (*39*). However, any errors in DFT propagate to MLFFs, reducing their reliability and predictive power (*40*–*42*). Given the critical role of charged NCIs across many applications and the growing use of MLFFs to extend DFT’s reach (*43*), it is imperative to address DFT’s deficiencies in describing these interactions.

In this work, we solve the DFT problem of charged NCIs by ensuring a balanced description of correlation effects in both neutral and charged systems through the integrative development of (r^2^SCAN+MBD)@HF. In (r^2^SCAN+MBD)@HF, the r^2^SCAN functional (*44*) and many-body dispersion (MBD) are evaluated on the Hartree-Fock (HF) orbitals, a combination essential for ensuring a consistent and accurate treatment of both neutral and charged NCIs. The r^2^SCAN functional was developed by Perdew and co-workers to overcome the numerical issues present in its predecessor, SCAN (*45*). On the other hand, evaluating approximate DFT functionals on HF orbitals has a long history in quantum chemistry (*46*, *47*), and, more recently, this procedure has been widely used in the context of density-corrected (DC) DFT, a formalism developed by Burke and co-workers to isolate density-driven errors in approximate DFT energies (*47*–*50*). Over the past decade, the same authors have identified many classes of systems for which evaluating approximate density functionals on HF orbitals yields substantially improved accuracy over self-consistent DFT (*18*).

Going back to (r^2^SCAN+MBD)@HF, beyond solving the problem of charged NCIs, our integrative design of (r^2^SCAN+MBD)@HF yields several key advantages over existing dispersion-enhanced DFT methods. (i) It achieves a balanced treatment of short- and long-range correlations across diverse NCIs, which is missed by other dispersion-enhanced DFT approximations. (ii) By using a nonempirical approach (the only MBD parameter is set to unity and is largely system insensitive), we avoid extensive empirical fitting of dispersion methods, which can be highly sensitive to the training set (*51*–*53*) and limit method transferability (*54*). (iii) Despite being designed to address the DFT deficiencies for charged NCIs, our method retains robust accuracy for neutral systems, matching or surpassing leading semilocal and hybrid functionals on main-group benchmarks. Furthermore, using HF densities within the “density- and dispersion-corrected DFT” framework of Sim and co-workers (*51*–*53*) serves a dual purpose in (r^2^SCAN+MBD)@HF: While HF densities typically improve upon approximate DFT densities for charged NCIs, their primary role here is to maintain balance between baseline DFT and MBD across different NCI types. Crucially, this balance is lost if DFT’s self-consistent density is used [e.g., if r^2^SCAN+MBD is used in place of (r^2^SCAN+MBD)@HF]. More broadly, altering any of its three components disrupts the consistent description of neutral and charged NCIs, typically leading to severe overbinding of charged complexes.

**Fig. 1.**
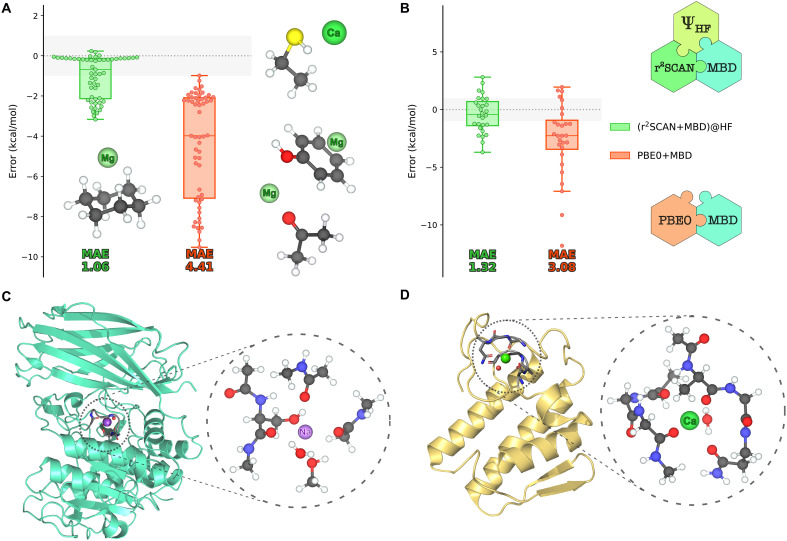
Interaction energy errors and representative structures for dication complexes in DES15K and MIPC datasets. Beeswarm and boxplots with the interaction energy errors [in kilocalories per mole (kcal/mol)] of (r^2^SCAN+MBD)@HF and PBE0+MBD methods for dication complexes in (**A**) DES15K and (**B**) MIPC datasets, with numbers at the bottom denoting the mean absolute errors (MAEs). Cartoon representations of proteins (**C**) 7O20 and (**D**) 1POD from the MIPC dataset, with zoomed-in views of their curated cluster structures. The labels for (C) and (D) correspond to Protein Data Bank (PDB) identifiers.

By addressing DFT’s deficiencies for charged NCIs while retaining accuracy for neutral systems, (r^2^SCAN+MBD)@HF provides a robust framework for quantum simulations of complex NCIs. The most challenging examples from our benchmark sets that include dications, ranging from small complexes to clusters extracted from our Metal Ion Protein Clusters (MIPC) dataset (see Results), are shown in Fig. 1 and illustrate major and consistent improvements of (r^2^SCAN+MBD)@HF over the widely used PBE0+MBD methods. As demonstrated in Results through the consistent success of (r^2^SCAN+MBD)@HF for charged NCIs, our method is ideally suited for computational studies in fields where charged NCIs are critical, including biomolecular interactions, adsorption in MOFs, and electrode materials in batteries.

## RESULTS

### (r^2^SCAN+MBD)@HF resolves charged NCI issues: DES15K insights

**Fig. 2.**
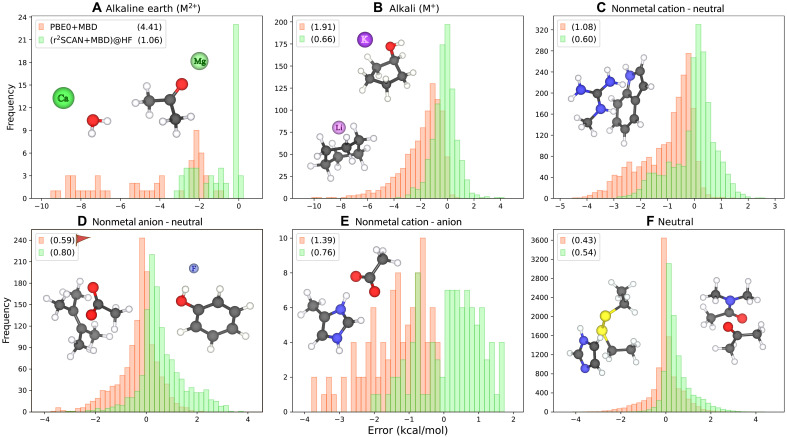
Error distributions for interaction energies across DES15K subsets. Histograms of error distribution for interaction energy predictions using (r^2^SCAN+MBD)@HF and PBE0+MBD methods for different subsets of DES15K dataset: (**A**) alkaline earth dication complexes, (**B**) alkali cation complexes, (**C**) nonmetal cation-neutral complexes, (**D**) nonmetal anion-neutral complexes, with the red flag indicating general and specific challenges of self-consistent DFT for anions (see text), (**E**) nonmetal cation-anion complexes, and (**F**) neutral complexes. Numbers in parentheses represent corresponding MAEs in kilocalories per mole (kcal/mol). Negative errors indicate overbinding, while positive errors indicate underbinding.

Figure 2 highlights a central finding of this work, demonstrating how (r^2^SCAN+MBD)@HF significantly improves dispersion-enhanced DFT accuracy for charged NCIs. We assess (r^2^SCAN+MBD)@HF on the DES15K dataset (*31*), which comprises 3052 unique dimers (distinct monomer pairs) and 14,651 dimer geometries, spanning both charged (approximately one-third) and neutral complexes (approximately two-thirds); see table S1 for the distribution across the interaction-type subsets. Each unique dimer in DES15K contains compressed, near-equilibrium, and slightly expanded geometries, sampled from potential energy surface scans and molecular dynamics simulations (*31*).

Specifically, Fig. 2 presents error distribution histograms for interaction energies in different DES15K categories, comparing (r^2^SCAN+MBD)@HF to the widely used PBE0+MBD. While many other DFT+Dispersion combinations could be considered, it is crucial to note that Johnson and co-workers have shown that dispersion methods systematically fail for charged NCIs (*17*), leading to large errors regardless of the method used. For this reason, we focus on comparing (r^2^SCAN+MBD)@HF with PBE0+MBD, which is not only representative of the performance of dispersion-enhanced DFT for charged NCIs (*17*) but is also a widely adopted in recent molecular dataset generation efforts (*55*, *56*).

Figure 2 (A and B) illustrates the most notable improvements of (r^2^SCAN+MBD)@HF for charged NCIs. For alkaline earth dication-neutral complexes (Fig. 2A), PBE0+MBD severely overbinds [mean absolute error (MAE) of 4.41 kcal/mol], while (r^2^SCAN+MBD)@HF reduces this error by a factor of four and yields much less skewed error distribution. Similarly, for alkali cation-neutral complexes (Fig. 2B), (r^2^SCAN+MBD)@HF reduces the MAE compared with PBE0+MBD (1.91 kcal/mol) by a factor of three, again resulting in a significantly less skewed error distribution. These results highlight (r^2^SCAN+MBD)@HF’s robustness in correcting DFT failures for NCIs with cations.

Figure 2C includes less polarizing nonmetal cations with more delocalized charges interacting with neutral molecules. Consequently, PBE0+MBD yields smaller errors (MAE of 1.08 kcal/mol) than in Fig. 2 (A and B). Yet, (r^2^SCAN+MBD)@HF still significantly improves accuracy, reducing the MAE nearly by half (to 0.60 kcal/mol), further demonstrating its robustness for charged NCIs.

We now turn to another DES15K subset: anion-neutral complexes. Figure 2D shows that both methods perform similarly for nonmetal anion-neutral pairs, with PBE0+MBD slightly outperforming (r^2^SCAN+MBD)@HF (MAE of 0.6 versus 0.8 kcal/mol). However, (r^2^SCAN+MBD)@HF is generally more robust for anion-containing NCIs because (i) semilocal DFT often yields unphysical results for anions with positive highest occupied molecular orbital energies that imply unbound states (*48*), hence the red flag next to PBE0+MBD in Fig. 2D; and (ii) for more challenging cases like the B30 dataset (*57*), (r^2^SCAN+MBD)@HF reduces the MAE by half compared with PBE0+MBD (table S10).

For cation-anion pairs (Fig. 2E), dominated by strong electrostatics, PBE0+MBD again significantly overbinds (MAE of 1.39 kcal/mol). (r^2^SCAN+MBD)@HF substantially improves accuracy (MAE of 0.76 kcal/mol), with most errors within ±2 kcal/mol. For neutral complexes (Fig. 2F), where PBE0+MBD already performs well (*17*), (r^2^SCAN+MBD)@HF remains comparably accurate (MAE of ∼0.5 kcal/mol).

Focusing on the large errors in DES15K interaction energies ( ∣error∣≥2 kcal/mol) and using symmetry-adapted perturbation theory (SAPT), which decomposes these interactions into electrostatic, induction, and dispersion components, we find another clear difference between the performance of the two methods. (r^2^SCAN+MBD)@HF exhibits only 559 such outliers (3.8% of DES15K), and these outliers are not only fewer in number but also confined to a much narrower physical regime, roughly matching the region where all the three SAPT components contribute comparably (see the SAPT ternary plots in fig. S1). In contrast, PBE0+MBD yields 1072 such cases with ∣error∣≥2 kcal/mol (7.3% of DES15K), spanning a broader physical regime wherever electrostatics or induction dominate.

These results demonstrate the robustness of (r^2^SCAN+MBD)@HF, highlighting its success in addressing dispersion-corrected DFT deficiencies for charged NCIs. In addition, despite being free from empirical parameters fitted to data, (r^2^SCAN+MBD)@HF matches the performance of modern dispersion-corrected semilocal and hybrid functionals on the diverse organic GMTKN55 dataset (table S11) (*58*), further emphasizing its robustness. In the following sections, we explore practical applications to metal-containing protein interactions and analyze the principles underlying (r^2^SCAN+MBD)@HF’s improved accuracy.

### (r^2^SCAN+MBD)@HF at work for metal-protein interactions

Having established the accuracy of (r^2^SCAN+MBD)@HF for DES15K, we now assess it on biologically relevant metal-protein interactions. To this end, we have curated the MIPC dataset, consisting of 45 biologically relevant metal-ligand complexes extracted from high-resolution (≤2.5 Å) Protein Data Bank (PDB) crystal structures with metal cations coordinated by both neutral and charged amino acid residues. The ≤2.5-Å resolution cutoff was applied because lower resolutions would risk poorly resolved ion-binding sites.

**Fig. 3.**
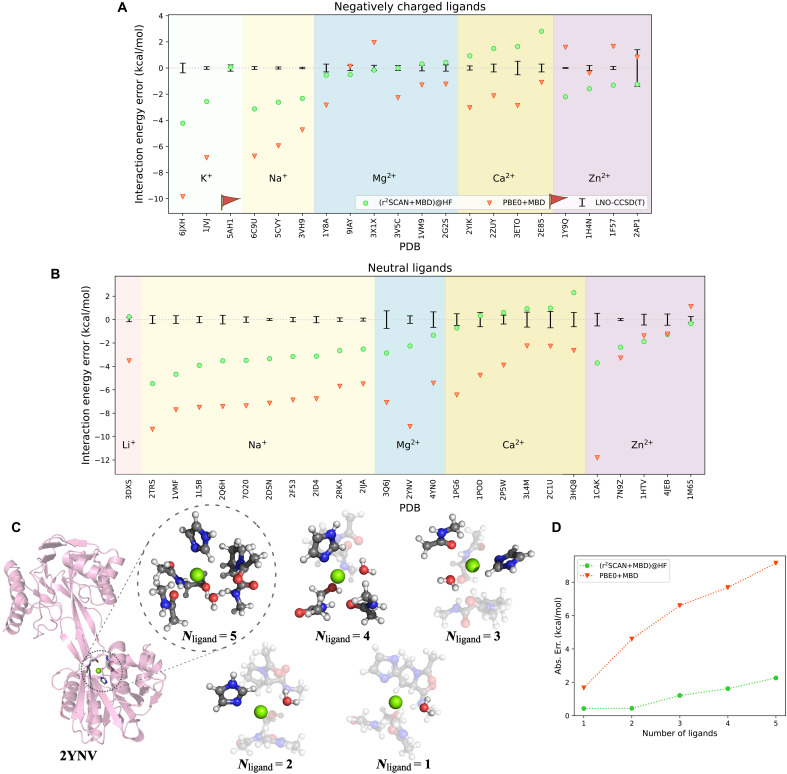
Interaction energy errors and subcluster analysis for MIPC complexes. Comparison of the interaction energy errors of (r^2^SCAN+MBD)@HF and PBE0+MBD methods for the MIPC dataset, including both subsets where the metallic cation is surrounded by (**A**) negatively charged and (**B**) neutral ligands, labeled by their PDB codes, with the red flag indicating the case for which the MBD part of PBE0+MBD could not be obtained (see text). Black error bars indicate uncertainty estimates of the LNO-CCSD(T) reference (see Materials and Methods). Representative protein structures (cartoon) and corresponding metal-ligand clusters (ball-and-stick) are shown in Fig. 1. (**C**) Subcluster generation for the Mg^2+^ cluster (PDB ID 2YNV): sequential ligand removal from the original cluster yielding subclusters with one to five coordinating ligands. (**D**) Absolute interaction energy errors (Abs. Err.) of (r^2^SCAN+MBD)@HF and PBE0+MBD relative to the LNO-CCSD(T) reference for each subcluster in (C).

In our MIPC, we distinguish a subset with negatively charged ligands (Fig. 3A) and a subset with neutral ligands (Fig. 3B). The former MIPC subset (Fig. 3A) includes important monovalent (Na^+^ and K^+^) and divalent (Mg^2+^, Ca^2+^, and Zn^2+^) ions in biochemistry. Mg^2+^ has a well-established catalytic role in phosphate chemistry (*59*). The most common superfamily of such enzymes is the P-loop containing nucleoside triphosphate hydrolases, so we included the highest resolution representatives containing Mg^2+^ with guanosine triphosphate (*60*) and guanosine diphosphate, separately (*61*). Zn^2+^ ions form zinc-fingers, an important motif for nucleic acid recognition (*62*). Typical coordinating residues are cysteins and histidines, we represented these by including Zn^2+^ sites selected for Zn-specific force-field development (*63*). We also included examples of Ca^2+^ clusters as Ca^2+^ is a very common cation in protein structures (*64*, *65*), with tightly regulated cellular concentrations and an important regulatory role in kinases (*66*).

The MIPC subset with neutral ligands (Fig. 3B) includes catalytic ion sites, e.g., the secondary Ca^2+^ site in phospholipase A_2_ (PDB ID 1POD) (*67*) or one of the Zn^2+^ ions of the zinc-cluster in the YcdX (PDB ID 1 M65), setting up a hydroxide nucleophile for phosphoesther hydrolysis (*68*). In other cases present in MIPC, the ion binding is linked to allosteric regulation of substrate binding (PDB IDs 2C1U and 7O20) (*69*, *70*). In an inhibitor screening study for sodium-coupled transporter LeuT (PDB ID 2Q6H) (*71*), sodium sites are in close proximity to the binding site of the leucine and the inhibitor clomipramine. The reliable computation of such binding energies is especially important when the identification of the bound cation relies on quantum-mechanical calculations (PDB ID 2DSN) (*72*).

For each protein in MIPC, we construct a cluster model by selecting amino acid residues within the first coordination shell of metal ions (Li^+^, Na^+^, K^+^, Mg^2+^, Ca^2+^, and Zn^2+^). We then benchmark the interaction energies between metallic cation and its surrounding amino acid residues using high-level local natural orbital (LNO)-CCSD(T) calculations (*12*, *13*, *73*, *74*) and include benchmark uncertainty estimates (see Materials and Methods).

Figure 3 compares (r^2^SCAN+MBD)@HF and PBE0+MBD errors against LNO-CCSD(T) reference interaction energies for MIPC (including uncertainty estimates), separating (A) negatively charged and (B) neutral ligands. The labels are PDB codes, and red flags mark negatively charged systems for which the MBD part of PBE0+MBD is not obtainable (see below). In Fig. 3 (A and B), the systems are grouped by metal cation and labeled by PDB codes, with representative proteins (cartoon) and corresponding metal-ligand clusters (ball-and-stick) from MIPC illustrated in Fig. 1.

For clusters with negatively charged ligands (Fig. 3A), our method achieves a MAE of 1.5 kcal/mol, while PBE0+MBD gives twice that value (see table S5 for individual cluster values). However, beyond the numerical improvements, our method is fundamentally better suited to systems involving negatively charged ligands than standard self-consistent hybrids such as PBE0. For one of the complexes in Fig. 3A, the MBD energies of PBE0+MBD cannot even be evaluated for the ligand part of the cluster (the K^+^-based cluster from PDB ID 5AH1, marked with a red flag), which contains negatively charged COO^−^ groups from two Asp residues. We can again trace back this failure to the deficiencies of standard self-consistent hybrid DFT for anions, which we discussed earlier (*48*, *50*, *75*). In the negatively charged ligand of the 5AH1 cluster, the PBE0 density errors distort the Hirshfeld volumes and produce unphysical atomic polarizabilities, which, in turn, lead to an unphysical MBD solution (*76*). Furthermore, the same type of PBE0+MBD failure observed for the 5AH1 cluster can also occur for other complexes in Fig. 3A when more diffuse basis sets are used (see fig. S9 for a concrete example involving the Zn^2+^-based cluster with PDB ID 2AP1). It is crucial to understand that these failures do not originate from the MBD model itself, but rather from erroneous PBE0 densities. Within our method, there is no problem with the MBD part for the 5AH1 cluster and the resulting (r^2^SCAN+MBD)@HF interaction energy also lies within the reference benchmark. In this way, by avoiding the fundamental limitations of PBE0 for anionic densities that can lead to the breakdown of MBD evaluated at those densities, our (r^2^SCAN+MBD)@HF approach is not only more accurate but also more physically grounded for the interaction energies of the protein clusters involving negatively charged ligands.

Moving now to Fig. 3B containing the MIPC clusters with neutral ligands, similar trends can be observed. Overall, (r^2^SCAN+MBD)@HF achieves a MAE of 2.3 kcal/mol, compared with 5.5 kcal/mol for PBE0+MBD (see table S6 for individual cluster values). For the single Li^+^ MIPC cluster [selected for therapeutic relevance (*77*)], the (r^2^SCAN+MBD)@HF error lies essentially within the reference uncertainty. For the Na^+^, Mg^2+^, and Ca^2+^ clusters, (r^2^SCAN+MBD)@HF shows slightly larger errors yet still substantially reduces the characteristic overbinding observed with PBE0+MBD. For the Zn^2+^ clusters in Fig. 3B, a closed-shell transition-metal cation not present in DES15K, both (r^2^SCAN+MBD)@HF and PBE0+MBD show small errors for most cases. However, for one Zn^2+^ cluster (PDB ID 1CAK), where PBE0+MBD exhibits pronounced overbinding, (r^2^SCAN+MBD)@HF significantly reduces the error.

While it has been reported in the literature that ion-water interaction energies computed with PBE0 combined with a dispersion method often exhibit good performance as the number of surrounding water molecules increases, it is useful to test how these errors evolve in the more enzyme-like coordination environments as those in our MIPC set. To examine this, we have analyzed the Mg^2+^ cluster (PDB ID 2YNV) already shown in Fig. 3B. Starting from the original cluster, we have removed one ligand at a time to generate five subclusters, in which the number of ligands varies from one to five (Fig. 3C). For each subcluster, we have benchmarked the interaction energy between Mg^2+^ and the present ligand(s), enabling us to assess how DFT errors behave as the number of ligands increases. The results in Fig. 3D show that, although the absolute error increases with the number of ligands for both PBE0+MBD and (r^2^SCAN+MBD)@HF, this increase is much less steep for (r^2^SCAN+MBD)@HF, indicating a more robust performance of (r^2^SCAN+MBD)@HF as the number of coordinating ligands around the ion increases.

MIPC represents a key step toward real-life systems also in terms of ion coordination: Ions in MIPC are coordinated by four to six ligands. This introduces several challenges for MIPC simulations. First, ion-ligand interaction errors can grow with the number of ligands, as illustrated in Fig. 3D. Second, modeling polarized ligand-ligand interactions is difficult as these interactions often become overly repulsive due to overpolarization in standard DFT methods. Third, as noted above, if negative charges are present in the ligand, then standard DFT can yield unphysical results. (r^2^SCAN+MBD)@HF effectively overcomes these issues, and the results from this section demonstrate that (r^2^SCAN+MBD)@HF reliably describes such complex charged interactions involving ions in protein or solvent environments. For example, these are critical to biological functions such as substrate binding, catalytic activity, and allosteric regulation. Its robust accuracy across diverse charged NCIs makes (r^2^SCAN+MBD)@HF a powerful method for biochemical simulations.

In contrast to the clusters discussed above involving metal cations, for the interaction energies of the glycine-water complexes of (*78*), PBE0+MBD yields slightly smaller errors than (r^2^SCAN+MBD)@HF (table S9). Nevertheless, the MAEs of both methods across the complexes with different forms of glycine (neutral, proton transfer transition state, and zwitterion) remain below 1 kcal/mol. At the same time, when comparing the four glycine protonation forms (negatively and positively charged, neutral, and zwitterionic) interacting with a single water molecule, (r^2^SCAN+MBD)@HF is slightly more accurate than PBE0+MBD (see fig. S16). For zwitterions, some caution is needed as standard hybrid DFT functionals, such as PBE0, have issues for zwitterionic systems, including unphysical charges and erratic SCF behavior. Thus, although (r^2^SCAN+MBD)@HF may exhibit accuracy comparable to or slightly below that of PBE0+MBD for zwitterionic complexes, it provides a physically more robust framework for treating these systems (*79*).

### The role of densities in (r^2^SCAN+MBD)@HF for cation-neutral pairs

Having demonstrated (r^2^SCAN+MBD)@HF’s accuracy for charged NCIs, we now analyze how it outperforms standard dispersion–enhanced DFT. A natural question arises: Is this success primarily due to the r^2^SCAN functional, the MBD term, or the use of HF densities? Analyzing the contributions of these components individually and combined, we find that (r^2^SCAN+MBD)@HF’s accuracy relies on their synergy: Altering any component disrupts this balance and compromises accuracy for charged and/or neutral NCIs.

We first examine the role of HF densities in (r^2^SCAN+MBD)@HF. It has been shown that evaluating functionals on the HF densities, instead of self-consistent ones, often significantly improves DFT accuracy across many systems (*18*, *48*, *50*, *52*, *53*). For certain systems (e.g., stretched heterodimers), HF densities are more accurate (i.e., energetically closer) to their exact counterparts than DFT ones, leading to DFT evaluated on HF densities improving over self-consistent DFT for the right reason (*80*). However, for other systems, such as transition states, whether this improvement for the right reason or error cancellations has been questioned (*81*, *82*).

**Fig. 4.**
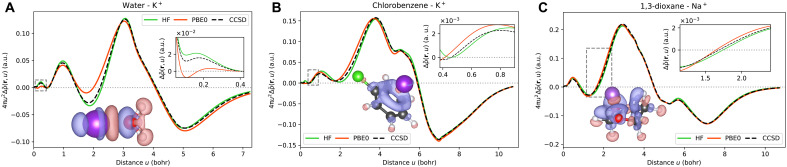
Deformation densities for representative cation-molecule complexes. (**A**) Spherically averaged deformation density, $\Delta \tilde{\rho }\left(r,u\right)$ (see Materials and Methods) for the $H_{2}O-K^{+}$ complex calculated using HF, PBE0, and CCSD methods, in which r placed at the position of the K^+^ nucleus. Inset shows a zoom-in of this quantity in the highlighted (boxed) u region. The other inset shows the standard deformation density, Δρ(r) , visualized as a three-dimensional isosurface (isovalue of 3.0 × 10^−4^ e/bohr^3^). (**B** and **C**) Same as in (A), but for chlorobenzene-K^+^ and 1,3-dioxane-Na^+^, respectively. a.u., atomic units.

Thus, to determine whether HF or DFT densities are more accurate for our systems of interest, we compare both against high-quality CCSD densities for representative cation-neutral complexes in Fig. 4. Specifically, for metallic cation-neutral molecule pairs, we analyze the spherically averaged deformation density, $\Delta \tilde{\rho }\left(r,u\right)$ , which isolates changes in electron distribution upon complex formation. Second, by placing r at the metal cation, $\Delta \tilde{\rho }\left(r,u\right)$ reveals how electron density redistributes, accumulating or depleting, around the metal cation as a function of radial distance *u*, providing a spherically averaged view of polarization effects. To define $\Delta \tilde{\rho }\left(r,u\right)$ , we first introduce the spherically averaged electron density, $\tilde{\rho }\left(r,u\right)$ (see Materials and Methods for a precise definition), which represents the average electron density at a distance u from a reference point r (*83*–*85*). Then, $\Delta \tilde{\rho }\left(r,u\right)$ is defined as the difference between $\tilde{\rho }\left(r,u\right)$ of the complex and the sum of those from its isolated fragments (the cation and the neutral molecule). $\Delta \tilde{\rho }\left(r,u\right)$ integrates to zero at every r , $\int_{0}^{\infty }\mathrm{du}\;4\pi u^{2}\Delta \tilde{\rho }\left(r,u\right)=0$.

Figure 4 shows $4\pi u^{2}\Delta \tilde{\rho }\left(r,u\right)$ for selected cation-neutral complexes computed at the CCSD, PBE0, and HF levels, with r placed at the cation. We also display CCSD isosurfaces of Δρ(r) (the usual deformation, i.e., interaction density) to visualize charge redistributions. For panels (A) (water - K^+^) and (B) (chlorobenzene - K^+^), the HF (spherically averaged deformation) densities more closely match CCSD results than PBE0, supporting their use in (r^2^SCAN+MBD)@HF. The results for more systems follow similar trends and are given in figs. S12 and S10. However, in panel (C) (1,3-dioxane-Na^+^), we cannot clearly determine whether HF or PBE0 is more accurate. While this analysis confirms that the HF (deformation) densities are more accurate than those of PBE0 for our cation-neutral complexes, it alone does not fully explain why (r^2^SCAN+MBD)@HF achieves its overall accuracy. Beyond improving density accuracy for charged NCIs, the HF densities also play a key role in (r^2^SCAN+MBD)@HF’s success by restoring the balance between the r^2^SCAN and MBD contributions to interaction energies, a critical aspect explored in the next section.

### The accuracy of (r^2^SCAN+MBD)@HF—The synergy of the three components

**Fig. 5.**
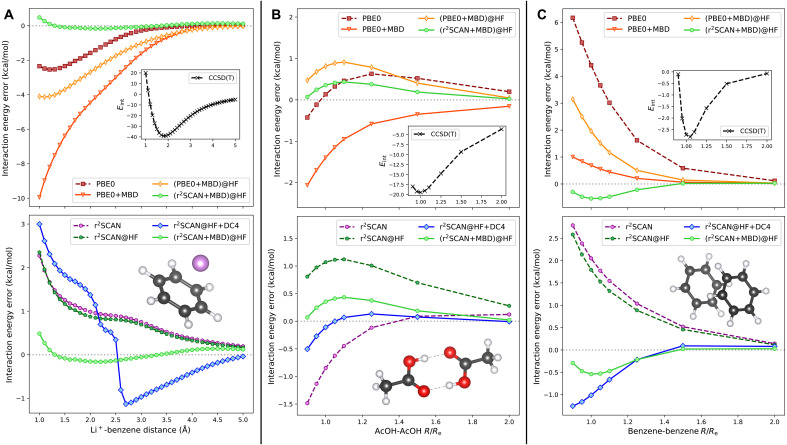
Errors of different DFT methods along the dissociation curves of representative complexes. Errors in computed interaction energies relative to CCSD(T) reference data (interaction curves shown as insets, top panels) for (**A**) Li^+^-benzene as a function of cation-benzene distance [in angstrom (Å)]; (**B**) acetic acid dimer; and (**C**) benzene dimer, each as a function of separation normalized to equilibrium distance ( $R/R_{e}$ ). Top panels show errors progressing from bare PBE0, adding MBD (PBE0+MBD), using DC version [(PBE0+MBD)@HF], to lastly (r^2^SCAN+MBD)@HF. Bottom panels similarly progress from bare r^2^SCAN, DC r^2^SCAN@HF, previously reported r^2^SCAN@HF+DC4, to (r^2^SCAN+MBD)@HF. As we progress along this sequence of functionals toward (r^2^SCAN+MBD)@HF, the interaction energy errors systematically decrease.

To demonstrate the synergy of its three components in achieving high accuracy for both neutral and charged NCIs, Fig. 5 shows dissociation curves for three prototypical NCI-bound complexes. In this figure, DFT methods are benchmarked against CCSD(T) references for (A) Li^+^-benzene (cation-neutral interaction), (B) acetic acid dimer (hydrogen bonded), and (C) benzene dimer (dispersion-bound π−π stacking). In Fig. 5, the top row compares (r^2^SCAN+MBD)@HF with PBE0-based variants, while the bottom row compares it with r^2^SCAN -based variants, highlighting that altering any of the three components in (r^2^SCAN+MBD)@HF compromises its accuracy.

An essential requirement for a base DFT functional is that it underbinds NCIs, allowing the dispersion term to properly compensate this underbinding [see (*37*) for a more formalized criterion using the concept of “dispersionless” functionals]. For example, PBE0 correctly underbinds the benzene dimer (Fig. 5C, top), allowing MBD to improve its binding. However, it already overbinds the Li^+^-benzene complex (Fig. 5A, top), so adding MBD only exacerbates the error. Thus, PBE0 fails to meet the fundamental requirement for dispersion-enhanced DFT: a base functional that consistently underbinds complexes across diverse NCIs, enabling dispersion corrections to accurately compensate.

On the other hand, self-consistent r^2^SCAN exhibits the desired underbinding behavior for the complexes in Fig. 5 (A and C, bottom), but not for the hydrogen-bonded acetic acid dimer in Fig. 5B (bottom), where it shows excessive overbinding, a behavior previously observed in (*52*) for hydrogen-bonded systems. Among the three base functionals used here, only r^2^SCAN evaluated on the HF density exhibits a consistent underbinding trend across the three prototypical systems for the three NCI types. The interaction energies are significantly improved once dispersion is restored through adding our MBD term to r^2^SCAN. This highlights the critical synergy among the three components (r^2^SCAN, MBD, and HF density) in (r^2^SCAN+MBD)@HF, enabling balanced accuracy across diverse NCIs, an essential feature not achieved by other combinations.

Now, we turn to the role of dispersion in (r^2^SCAN+MBD)@HF, which is more subtle. Previously, r^2^SCAN@HF combined with the D4 correction and DC parameters, yielding r^2^SCAN@HF-DC4, achieved excellent general accuracy, especially for hydrogen-bonded complexes and water (*52*). Consistent with this, r^2^SCAN@HF-DC4 performs well for neutral complexes (Fig. 5, B and C, bottom) but notably fails for the Li^+^-benzene system (Fig. 5A, bottom). The reparametrized version of r^2^SCAN@HF-DC4 based on the dual-calibration approach (*53*) reduces the error at intermediate and long Li^+^-benzene separations, yet the underlying erroneous behavior, including an unphysical jump in the dissociation curve, remains (fig. S7). This failure arises because of the sensitivity of the r^2^SCAN@HF-DC4 results to both the density and its D4 empirical parameters (*51*, *52*) for the cation-neutral pairs. To elaborate on this, we have to go into more technical details on the construction of the dispersion methods. Namely, both MBD and D4 rely (at least indirectly) on the density information, with the latter requiring partial atomic charges for its construction (*33*). By default settings, classical partial atomic charges trained on the DFT density data are used in D4 (*33*). Because these settings are used in both the training and application of r^2^SCAN@HF-DC4, this method still retains input from the DFT densities, although it is designed for the HF densities. On top of the indirect DFT density input, the classical charges used in the D4 part of r^2^SCAN@HF-DC4 are based on the so-called electronegativity equilibration model, which inherently suffers from artificial long-range charge transfer, especially for systems containing ions (*86*). In contrast to these problems present in r^2^SCAN@HF-DC4, (r^2^SCAN+MBD)@HF strictly computes both the functional and the MBD term on the HF densities without any input from the DFT densities. Moreover, (r^2^SCAN+MBD)@HF is nearly nonempirical, with its only empirical parameter β , which controls the range-separation of MBD interaction, set to unity. Crucially, when coupled with r^2^SCAN@HF, MBD@HF exhibits remarkable insensitivity to β variations across different systems, as we will show later.

### Transforming r^2^SCAN@HF’s initial weakness into (r^2^SCAN+MBD)@HF’s key strength

**Fig. 6.**
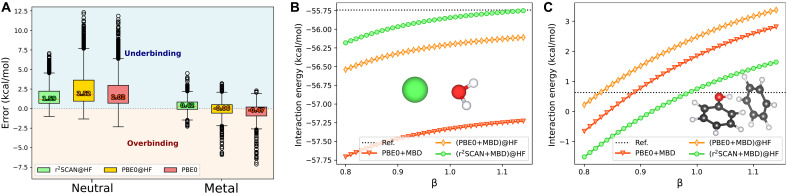
Systematic underbinding of r^2^SCAN@HF and MBD-parameter sensitivity of interaction energies. (**A**) Boxplot of interaction energy errors [in kilocalories per mole (kcal/mol)] for r^2^SCAN@HF, PBE0@HF, and PBE0 across neutral and metal subsets of DES15K. Boxes show interquartile ranges, medians (lines), outliers (circles), and mean errors (numbers). (**B** and **C**) Dependence of DFT+MBD interaction energies on the MBD parameter β for (B) $H_{2}O-Ca^{2+}$ and (C) benzene-phenol complexes. Reference interaction energies [CCSD(T)] are indicated by dotted lines.

In Fig. 2, we have shown that (r^2^SCAN+MBD)@HF success for charged NCIs is due to the complementarity between MBD and r^2^SCAN@HF, with MBD accurately compensating for r^2^SCAN@HF’s systematic underbinding. This ensures balanced accuracy across both charged and neutral NCIs, a key advantage of (r^2^SCAN+MBD)@HF over other dispersion-enhanced methods. What enables this balance is the consistent underbinding behavior of r^2^SCAN@HF, as observed previously for prototypical complexes across different NCIs. Here, we further demonstrate that this critical behavior of r^2^SCAN@HF, essential for (r^2^SCAN+MBD)@HF’s success (Figs. 2, 3, and 5), is systematic, using the broader DES15K dataset. Figure 6A compares signed interaction energy errors from r^2^SCAN@HF, PBE0@HF, and PBE0 across neutral (left) and metal cation-neutral complexes (right). Only r^2^SCAN@HF consistently underbinds both neutral and cation complexes, unlike PBE0-based methods. As a result, only r^2^SCAN@HF, among the three methods, can be transformed into an accurate dispersion-enhanced DFT approach by adding a dispersion term. In this way, the systematic underbinding by r^2^SCAN@HF, initially a weakness, becomes (r^2^SCAN+MBD)@HF’s key strength when combined with MBD, enabling balanced accuracy for charged and neutral NCIs.

### Completing the puzzle: How MBD and r^2^SCAN enable (r^2^SCAN+MBD)@HF’s accuracy

Now, we examine the final piece of the puzzle–MBD’s role in (r^2^SCAN+MBD)@HF’s success. MBD has only one empirical parameter, β , which we set to unity in (r^2^SCAN+MBD)@HF. Increasing β weakens MBD contribution to interaction energies, vanishing as β→∞. Figure 6B shows how the interaction energy for the $H_{2}O-Ca^{2+}$ complex depends on the MBD parameter β for r^2^SCAN@HF, PBE0, and PBE0@HF, with the reference energy indicated by the horizontal dashed line. For (r^2^SCAN+MBD)@HF (green curve), the optimal β is slightly above 1, making our choice of β = 1 nearly optimal. In contrast, because PBE0 and PBE0@HF already overbind the complex, their β-curves never cross the reference line, instead favoring large β values (ideally, β→∞ ) to suppress further overbinding from MBD.

A stark contrast appears in Fig. 6C for the neutral benzene-phenol complex. Here, PBE0 and PBE0@HF require smaller β values (0.8 to 0.9) to match the reference, unlike the large β values favored previously (Fig. 6B). (r^2^SCAN+MBD)@HF again aligns closely with the reference at β≈1 , highlighting its excellent consistency across charged and neutral NCIs.

Further analysis in the Supplementary Materials, covering additional systems (fig. S15) and transferability tests of DFT+MBD trained on neutral NCIs and evaluated on charged NCIs and vice versa (fig. S14), confirms the results of Fig. 6 (B and C). Only when using r^2^SCAN@HF as a baseline does DFT+MBD exhibit consistently small sensitivity to β across different NCIs, allowing (r^2^SCAN+MBD)@HF to simultaneously maintain high accuracy for both neutral and charged interactions. In contrast, the greater sensitivity of other methods implies improving accuracy for one interaction type inevitably reduces it for the other. Thus, (r^2^SCAN+MBD)@HF’s unique β-insensitivity makes it ideal for simulations of systems where different interaction types coexist, such as biomolecular complexes (e.g., Fig. 3).

To further motivate the choice of r^2^SCAN in our (r^2^SCAN+MBD)@HF, we have tested other functionals within the (DFT+MBD)@HF methodology for DES15K categories. To enable efficient comparison across many functionals, we have used a genetic algorithm (GA) to construct representative subsets of the metal-containing and neutral DES15K categories. These subsets have been selected such that they reproduce the error statistics of the full DES15K categories for the DFT methods on which we performed full-set calculations (fig. S2). These GA-selected subsets have enabled us to cost-effectively test a larger number of functionals for neutral and metal-containing DES15k complexes and also to test higher-level methods (see below). Testing different functionals, such as M06 (*87*), TPSS (*88*), and B3LYP, within the (DFT+MBD)@HF methodology for both GA-selected neutral and metal-containing DES15K complexes confirms that (r^2^SCAN+MBD)@HF delivers the best overall performance (fig. S3). It is followed by other SCAN-family members [SCAN (*45*) and r^2^SCAN0 (*89*)] and then by (M06+MBD)@HF. Additional tests of (M06+MBD)@HF, such as those on our MIPC and on paradigmatic cases of Fig. 5, show that (r^2^SCAN+MBD)@HF performs substantially better across diverse NCIs than (M06+MBD)@HF (figs. S5 and S7, respectively), despite the former being free of parameters fitted to data.

In terms of computational cost, (r^2^SCAN+MBD)@HF is comparable to hybrid functionals, because it combines semilocal r^2^SCAN with an HF calculation, resulting in an overall cost similar to PBE0+MBD. To place our results in the broader context of DFT accuracy and cost, we have compared (r^2^SCAN+MBD)@HF with representative semilocal, hybrid, range-separated hybrid, and double-hybrid methods on three DES15K categories: the alkaline earth subset and the GA-selected alkali and neutral subsets. As a semilocal (lowest cost), we have included r^2^SCAN+D4 (*33*, *44*), while, at the hybrid level, we have used PBE0+MBD and the (DFT+MBD)@HF variants mentioned earlier. At a higher cost than standard hybrids, we have selected ωB97M-V (*90*), the range-separated hybrid with nonlocal correlation [see fig. S4 for a direct wall-time comparison with (r^2^SCAN+MBD)@HF on a representative MIPC cluster]. Last, we have included revDSD-PBEP86-D4 (*91*) as a representative double hybrid, the most computationally costly DFT class. ωB97M-V and revDSD-PBEP86-D4 were chosen because they were used in the literature to effectively model NCIs (*78*). Across the three DES15K categories considered (alkaline earth, alkali, and neutral), (r^2^SCAN+MBD)@HF is the only method among all tested DFT approaches that achieves chemical accuracy (MAEs below 1 kcal/mol) simultaneously across all three categories, as shown in fig. S4. Notably, ωB97M-V fails to achieve this accuracy for the alkaline earth subset, and revDSD-PBEP86-D4 does not achieve it for the alkali subset (see also fig. S6 for the corresponding error-distribution boxplots). These tests highlight the advantage of (r^2^SCAN+MBD)@HF even relative to more costly DFT methods for charged NCIs.

## DISCUSSION

Accurate yet tractable quantum-mechanical treatment of charged NCIs remains a key challenge with broad implications from biochemistry to materials. Dispersion-enhanced DFT has major difficulties in describing charged NCIs due to the interplay of density errors, coupling of polarization and dispersion, and the poor transferability of dispersion corrections trained on neutral systems. Improving charged NCIs’ accuracy without compromising neutral ones cannot be solved by merely adding charged systems to the dispersion method training. Instead, as we show here, it requires a foundational solution that balances correlation effects for both neutral and charged NCIs. Specifically, achieving this balance requires a dispersion method to accurately compensate for what the base DFT misses, which is easier for neutral NCIs but becomes highly nontrivial for charged ones. Restoring this balance is central to the integrative design of (r^2^SCAN+MBD)@HF for accurate treatment of both charged and neutral NCIs. Crucially, altering any of its three components (r^2^SCAN, MBD, or HF densities) breaks this balance.

A distinguishing feature of (r^2^SCAN+MBD)@HF is its overall minimal empiricism, (i.e., it is free from empirical parameters fitted to data) reflected in each of its three components. Despite this, (r^2^SCAN+MBD)@HF improves accuracy for charged NCIs, even relative to higher level DFT approaches, such as ωB97M-V and revDSD-PBEP86-D4. It also maintains performance for neutral NCIs and achieves accuracy comparable to leading semilocal functionals on main-group benchmarks (GMTKN55). This broader accuracy enables (r^2^SCAN+MBD)@HF to support applications from biochemistry to materials, where charged NCIs coexist with other interactions. For example, in biochemistry, accurate simulations of enzymatic reactions with a metallic cation in the active site require capturing both cation affinity and reaction kinetics (*92*, *93*). These challenges often couple when cations stabilize transition states, as with Mg^2+^ in kinase-catalyzed phosphoryl transfers (*92*, *93*). Thus, describing such systems requires a method that can capture both reaction barriers and NCIs involving the cation, making (r^2^SCAN+MBD)@HF a strong candidate for advancing enzymatic simulations. This is strongly supported by our MIPC results, which demonstrate our method’s high accuracy for metal-protein interactions, including cases where the metal ion is surrounded by negatively charged ligands for which standard DFT can completely break down.

On the materials side, an example of interactions between metallic cations and neutral gases is the challenging adsorption of small molecules on open-metal-site (OMS) MOFs (*94*), which is another target for future applications of (r^2^SCAN+MBD)@HF. These systems are hard to simulate as classical force fields are unreliable near the OMS (*95*), and the recently developed MLFFs for MOFs display limitations due to the DFT training data (*96*). We expect that this problem can be addressed by training MLFFs on (r^2^SCAN+MBD)@HF data. More broadly, (r^2^SCAN+MBD)@HF can serve as a general-purpose DFT method for generating high-quality reference data to train MLFFs, particularly for charged systems, with (r^2^SCAN+MBD)@HF’s forces implemented following (*97*, *98*).

## MATERIALS AND METHODS

### DFT calculations

All DFT calculations were performed using pySCF package (*99*), whereas ωB97M-V and double-hybrid calculations were performed with ORCA 5.0.4 (*100*). For all calculations except MIPC, we used def2-QZVPPD basis set (*101*) and def2-TZVPD basis set (*101*) for MIPC calculations. In all Orca calculations, RIJCOSX approximation was used with corresponding auxiliary basis sets (*102*) to accelerate the calculations, TightSCF was used as SCF convergence threshold, and DEFGRID2 was used as DFT grid. In double-hybrid calculations, def2-QZVPPD/c auxiliary basis sets were also used to accelerate RI-MP2 calculations. The base functional r^2^SCAN@HF and PBE0@HF energies were evaluated on converged HF densities. The default grid level 5 in pySCF was used for all HF-DFT calculations. The MBD calculation were performed on converged HF and PBE0 densities using libMBD package (*103*). The empirical range-separation parameter β = 1.0 in MBD@rsSCS (*27*) was selected for all calculations, unless otherwise specified. The high-level reference data for Li^+^-benzene dissociation curve were reported in (*17*). The reference data of other dissociation curves in Fig. 5 are from S66x8 database (*104*).

Spherically averaged density that we used in Fig. 4 is defined as

$$\tilde{\rho }\left(r,u\right)=\frac{1}{4\pi }\int_{0}^{\pi }\int_{0}^{2\pi }\rho \left(r+u\right)\text{sin}\phi \;d\theta \;d\phi$$

and it satisfies

$$4\pi \int_{0}^{\infty }\tilde{\rho }\left(r,u\right)\;u^{2}\;\mathrm{du}=N$$

where N is the number of electrons. The integrand $4\pi u^{2}\tilde{\rho }\left(r,u\right)$ is defined as radial density.

Spherically averaged deformation density is defined as

$$\Delta \tilde{\rho }\left(r,u\right)={\tilde{\rho }}_{\text{AB}}\left(r,u\right)-{\tilde{\rho }}_{A}\left(r,u\right)-{\tilde{\rho }}_{B}\left(r,u\right)$$

and

$$4\pi \int_{0}^{\infty }\Delta \tilde{\rho }\left(r,u\right)\;u^{2}\;\mathrm{du}=0$$

### Cluster creation

For the MIPC clusters in Fig. 3A containing negatively charged ligands, we have sourced Ca^2+^ and Mg^2+^ binding proteins from the nonredundant ccPDB 2.0 dataset (*105*), while the clusters including Zn^2+^, Na^+^, and K^+^ have been sourced from ion-specific classical force field development studies (*63*, *106*). Table S2 summarizes the dataset sources, chosen to limit redundancy and to make use of literature studies on metal-binding sites. From the initial cluster set, we excluded clusters with fewer than four ligands surrounding the metal cation. For the final MIPC subset with negatively charged ligands (Fig. 3A), we have used the highest-resolution representative for the most common ion-coordination profiles.

Our second MIPC cluster set of Fig. 3B is restricted to only contain neutral ion-binding residues, which reduces the available structures significantly. We queried the RCSB PDB for structures with resolution of ≤2.5 Å as of 26 July 2024 and then filtered out sites that contained charged residues within 5 Å of the metal ion. These criteria reduced the structure pool to 65 sites, further decreasing to 36, omitting those that have a coordination number less than 4. Twenty-six nonredundant structures were manually selected to minimize structural redundancy.

The MIPC protein structures have been extracted from PDB and then completed with explicit hydrogen atoms by using CHARMM-GUI (*107*), relying on the HBUILD utility (*108*). Structures were used as is, without minimization. To create protein-ion clusters (i.e., cation with surrounding amino acid residues), we have made an initial selection of atoms within 3.5 Å of the ion. We then inspected atoms covalently bonded to this initial selection and decided, for each bond, whether the bonded atom should be included in the cluster or whether the bond should be cut. Bond cutting was allowed only at specific positions, namely, at single bonds between two sp^3^ carbon atoms (and in rare cases where this was not possible, at the C_α_─N backbone bond). Any cut bond was capped with a hydrogen atom to produce the final cluster. The algorithm used for this purpose has been made publicly available on GitHub: https://github.com/bertadenes/qm_selector.

### LNO-CCSD(T) reference interaction energies

CCSD(T) reference calculations were performed with our linear-scaling LNO-CCSD(T) (*12*, *13*, *73*, *74*) method in the MRCC (*109*–*111*) program suite. To accelerate the convergence toward the complete basis set (CBS) limit, we combine basis set extrapolation, counterpoise correction (*112*), and density-based basis set correction (DBBSC) (*113*). The CBS and local approximation free (LAF) limit of CCSD(T) is estimated for the MIPC set as

$$E_{\text{CCSD}\left(T\right)}=E_{N-T\;\text{LNO}}^{\text{TZ},\text{DBBSC}}-E_{\text{Normal}\;\text{LNO}}^{\text{TZ},\text{DBBSC}}+E_{\text{Normal}\;\text{LNO}}^{\text{CBS}\left(T,Q\right),\text{DBBSC}}$$ (1)

Here, superscripts *X*Z refer to the cardinal number *X* of the aug-cc-pV(*X* + *d*)*Z* basis sets (*114*), while the corresponding CBS extrapolated values are denoted as CBS(*X*,*X* + 1) (*115*). Subscripts Normal (N) and Tight (T) denote LNO thresholds, while N–T denotes LAF extrapolation toward canonical CCSD(T) using Normal and Tight LNO settings (*12*, *13*). The LAF extrapolation in the first term is carried out via $E_{N-\text{TLNO}}^{\text{TZ},\text{DBBSC}}=E_{\text{TightLNO}}^{\text{TZ},\text{DBBSC}}+0.5\left(E_{\text{TightLNO}}^{\text{TZ},\text{DBBSC}}-E_{\text{NormalLNO}}^{\text{TZ},\text{DBBSC}}\right)$ , following the formula explained in (*12*) and (*13*). The system-specific uncertainty estimates corresponding to Eq. 1, plotted in Fig. 3, are the sum of uncertainty estimates for the basis set and LNO approximations. These are obtained, respectively, as the size of the DBBSC correction at the CBS(T,Q) level and via the LAF framework (*12*, *13*) as $\pm 0.5(E_{\text{Tight}\;\text{LNO}}^{\text{TZ},\text{DBBSC}}-E_{\text{Normal}\;\text{LNO}}^{\text{TZ},\text{DBBSC}})$ . This level of convergence provides, on the average, about ±0.5 kcal/mol uncertainty for the LNO-CCSD(T) interaction energies of the MIPC set. An additional benefit of the composite Eq. 1, in addition to its robust and low uncertainty estimate, is its computational cost. The required LNO-CCSD(T) computations took about 1 to 2 days of wall time with few tens of CPU cores and ~50 GBs of minimal memory requirement for the complexes of 40 to 70 atoms. LNO-CCSD(T) for the largest, 90 to 110 atom complexes required about twice as much resource. The reliability of the CCSD(T) estimate of Eq. 1 is extensively validated against even better converged LNO-CCSD(T) results for five representative protein-ion complexes (see the Supplementary Materials for additional details).

## Supplementary Materials

### The PDF file includes:

Supplementary Materials and Methods Figs. S1 to S21 Tables S1 to S11 Legends for data S1 to S4 References
