# Supplementary material for: Accurate density functional theory for noncovalent interactions in charged systems
Source: Sci Adv. 2026 Apr 22;12(17):eadz8521. doi: 10.1126/sciadv.adz8521 (PMC13101883; doi:10.1126/sciadv.adz8521)
Supplement: Supplementary file 1 — Supplementary Materials and Methods Figs. S1 to S21 Tables S1 to S11 Legends for data S1 to S4 References [file sciadv.adz8521_sm.pdf]

Science Advances
AAAS

Supplementary Materials for

Accurate density functional theory for noncovalent interactions in charged systems

Heng Zhao *et al.*

Corresponding author: Stefan Vuckovic, stefan.vuckovic@unifr.ch

Sci. Adv. **12**, eadz8521 (2026)
DOI: 10.1126/sciadv.adz8521

The PDF file includes:

Supplementary Materials and Methods
Figs. S1 to S21
Tables S1 to S11
Legends for data S1 to S4
References

Other Supplementary Material for this manuscript includes the following:

Data S1 to S4

Materials and Methods

LNO-CCSD(T) reference interaction energies

A detailed basis set and LNO convergence study is presented to verify the accuracy of the LNO-CCSD(T) reference MIPC interaction energies. To that end, 5 representative complexes are selected covering the investigated cation types and exhibiting the highest deviation between (r^2SCAN+MBD)@HF and PBE0+MBD. Namely, we considered 2TRS for Na^{+}, 2YNV for Mg^{2+}, 1PG6 for Ca^{2+}, as well as 1CAK and 7N9Z for Zn^{2+} (see Figs. S17- S21).

Regarding the local approximations in the LNO-CCSD(T) method (*12, 13, 73, 74, 116*) the systematically improving series of `Normal` (N), `Tight` (T), `very Tight` (vT), and `very very Tight` (vvT) LNO thresholds were employed. To improve the convergence towards the local approximation free (LAF) limit of CCSD(T), LAF extrapolations (*12, 13*) were carried out using the above LNO threshold series, yielding the N–T, T–vT, and vT–vvT LAF extrapolated LNO-CCSD(T) results.

These LNO-CCSD(T) calculations employed the aug-cc-pV(X+d)Z (*114*) basis sets (X = D, T and Q) on the first and second row elements, with the respective weighted core-valence aug-cc-pwCVXZ (*117, 118*) bases used for the metal ions to account for their subvalence correlation. For Ca^{2+} and Zn^{2+}, we utilized the pseudopotential-based aug-cc-pwCVXZ-PP basis sets, with MCDHF-ECP-10 effective core potentials (*119*). We performed CBS extrapolations on the HF (*120*) and correlation (*115*) energies [CBS(X,X + 1)] to further accelerate the basis set convergence. Besides counterpoise corrections (*112*), we added complementary auxiliary basis set (CABS) to the HF and the recent LNO-based (*113*) density-based basis set correction (DBBSC) (*121*) to the CCSD(T) correlation energies.

Using the best converged LNO and basis set settings, our most accurate composite CCSD(T)/CBS estimate is:

$$E_{\mathrm{CCSD(T)+}} = E^{\mathrm{TZ,DBBSC}}_{\mathrm{T-vT\ LNO}} - E^{\mathrm{TZ,DBBSC}}_{\mathrm{Tight\ LNO}} + E^{\mathrm{CBS(T,Q),DBBSC}}_{\mathrm{Tight\ LNO}}, \qquad \text{(S1)}$$

which is labeled with a golden cross on Figs. S17- S21. The uncertainty estimate corresponding to this $E_{\mathrm{CCSD(T)+}}$ estimate is obtained as the sum of uncertainty estimates for the basis set and LNO approximations. These are obtained, respectively, as the size of the DBBSC correction at the

CBS(T,Q) level and via the LAF framework (*12, 13*) as $\pm 0.5(E^{\text{TZ,DBBSC}}_{\text{veryTight LNO}} - E^{\text{TZ,DBBSC}}_{\text{Tight LNO}})$:

$$E_{\text{CCSD(T)+,uncertainty}} = \frac{1}{2}\left|E^{\text{TZ,DBBSC}}_{\text{vTight LNO}} - E^{\text{TZ,DBBSC}}_{\text{Tight LNO}}\right| + \frac{1}{2}\left|E^{\text{CBS(T,Q),DBBSC}}_{\text{N-T LNO}} - E^{\text{CBS(T,Q)}}_{\text{N-T LNO}}\right| \qquad \text{(S2)}$$

The detailed convergence study for the five cases (Figs. S17- S21) show excellent level of agreement with the `vTight` and `vvTight` thresholds. For example, uncertainties of ±0.10 kcal/mol and ±0.01 kcal/mol are reached at the vT–vvT LAF extrapolated level for the 2YNV and 7N9Z complexes (cf. green aug-cc-pV(T+d)Z curves of Figs. S17- S21). Importantly, the T–vT LAF extrapolated results are very close to vT–vvT (within ca. ±0.1 kcal/mol), and even the N–T LAF extrapolations exhibit an uncertainty of at most ±0.3–0.4 kcal/mol.

Regarding the basis set convergence, we find excellent agreement between the DBBSC-corrected and uncorrected results with the larger basis sets and CBS extrapolation. Especially at the CBS(T,Q) level (dark orange curves of Figs. S17- S21), their agreement is mostly within ca. ±0.1 kcal/mol and always better than ±0.3 kcal/mol. While the CBS(D,T) and CBS(T,Q) results are often close, our uncertainty measure, i.e. the size of the DBBSC correction is larger at the CBS(D,T) level (purple curves of Figs. S17- S21). Thus, aiming for higher accuracy and lower uncertainty estimates we invest in the quadruple-ζ computations needed for CBS(T,Q). While, if a bit higher uncertainty is tolerable, the more affordable and memory-economic CBS(D,T) could be recommended, as long as it is counterpoise and DBBSC corrected. All in all, about ±0.3 kcal/mol uncertainty [Eq. (S2)] is assigned to $E_{\text{CCSD(T)+}}$ of Eq. (S1) this way.

On the basis of analyzing Figs. S17- S21, an even more affordable composite energy expression will be used for the remaining entries of the MIPC set [while Eq. (S1) is retained when available):

$$E_{\text{CCSD(T)}} = E^{\text{TZ,DBBSC}}_{\text{N-T LNO}} - E^{\text{TZ,DBBSC}}_{\text{Normal LNO}} + E^{\text{CBS(T,Q),DBBSC}}_{\text{Normal LNO}} \,. \qquad \text{(S3)}$$

This $E_{\text{CCSD(T)}}$ (light blue cross on Figs. S17- S21) is very close to the tightly converged $E_{\text{CCSD(T)+}}$, their difference is mostly below 0.1 kcal/mol, and only up to 0.3 kcal/mol for the worst case of 1cak. Moreover, the uncertainty estimates of $E_{\text{CCSD(T)}}$ fully envelope the uncertainty estimates of $E_{\text{CCSD(T)+}}$, highlighting their consistency.

The benefit of $E_{\text{CCSD(T)}}$ it that it spares the most demanding `vTight` LNO-CCSD(T)/TZ and `Tight` LNO-CCSD(T)/QZ computations needed for Eq. (S1), thus it is about an order of magnitude faster and 4-fold more memory economic. For the sake of completeness, we note the use of frozen-core approximation (with correlated subvalence metal ion orbitals), conventional auxiliary basis

sets (*102, 122*), and core-valence auxiliary basis sets (*123, 124*) for all LNO-CCSD(T) interaction energies.

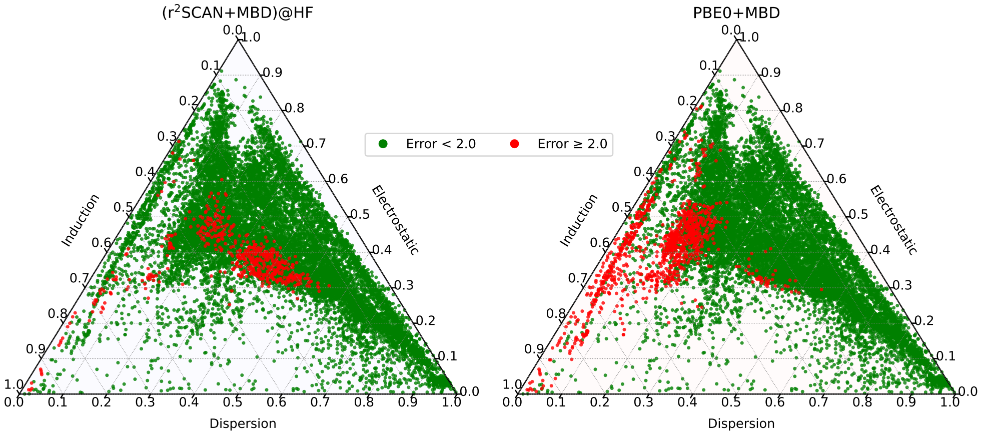

Figure S1: **Ternary SAPT energy decomposition and outliers of two methods in the DES15K dataset.** Ternary plots of the whole DES15K dataset, showing the relationship between the electrostatic, dispersion, and induction energy components from symmetry-adapted perturbation theory (SAPT) calculations taken from ref. (*31*), with red dots showing the outliers (Error ≥ 2 kcal/mol) of the (r^2SCAN+MBD)@HF (left) and PBE0+MBD methods (right).

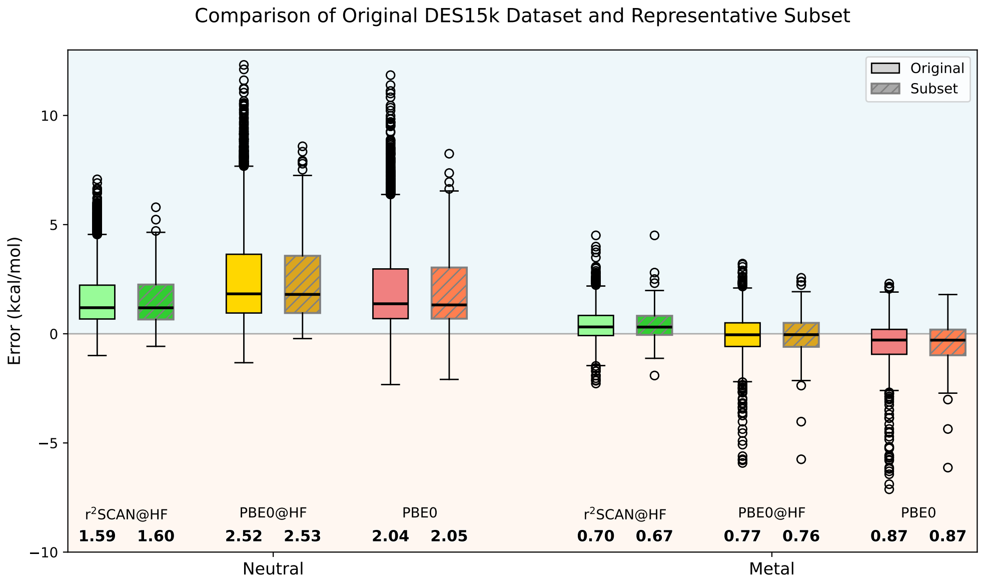

Figure S2: **Creating GA-selected subsets of the Neutral and Metal categories of DES15K.** Error distributions for selected density functionals on the original DES15K categories and on the corresponding genetic algorithm (GA)-selected subsets. Within each category, boxes are grouped by functional, with "Orig" indicating the original DES15K dataset and "Subset" indicating the GA-selected subset. The GA was used to select each "Subset" such that its error distributions closely mimic the "Orig" error distributions for the two categories ("Neutral" for neutral complexes and "Metal" for the metal-containing complexes). For the Neutral category, the "Orig" set contains 10263 complexes, and the corresponding "Subset" (GA-selected) contains 200 complexes. For the Metal category, the "Orig" set contains 958 complexes (903 alkali and 55 Alkaline earth), and the corresponding "Subset" (GA-selected) contains 100 complexes (97 alkali and 3 Alkaline earth). Box plots show the distribution of interaction energy errors (kcal/mol) relative to reference values. Median values are marked by thick black lines, and mean absolute errors (MAEs) are displayed below each pair of boxes. A complete list of the complexes contained in each GA-selected "Subset" is provided in Supplementary Data D1".

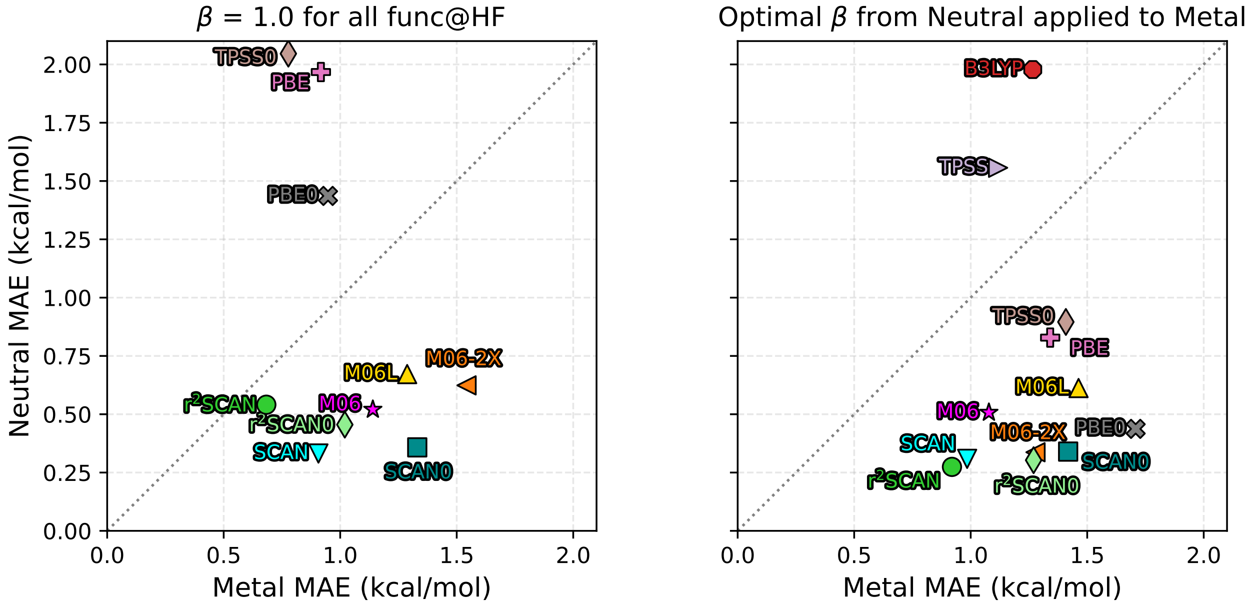

Figure S3: **Performance of (DFT+MBD)@HF functionals on GA-selected DES15K subsets.** Parity plots of mean absolute errors (MAEs) for GA-selected neutral and metal subsets from DES15K dataset (see Fig. S2 for the selection of these GA-selected subsets), computed with (DFT+ MBD)@HF methods with various functionals. In the left panel, the empirical parameter β of MBD is fixed as 1.0 for all functionals; in the right panel, β is tuned for the best performance of individual functional on Neutral subset.

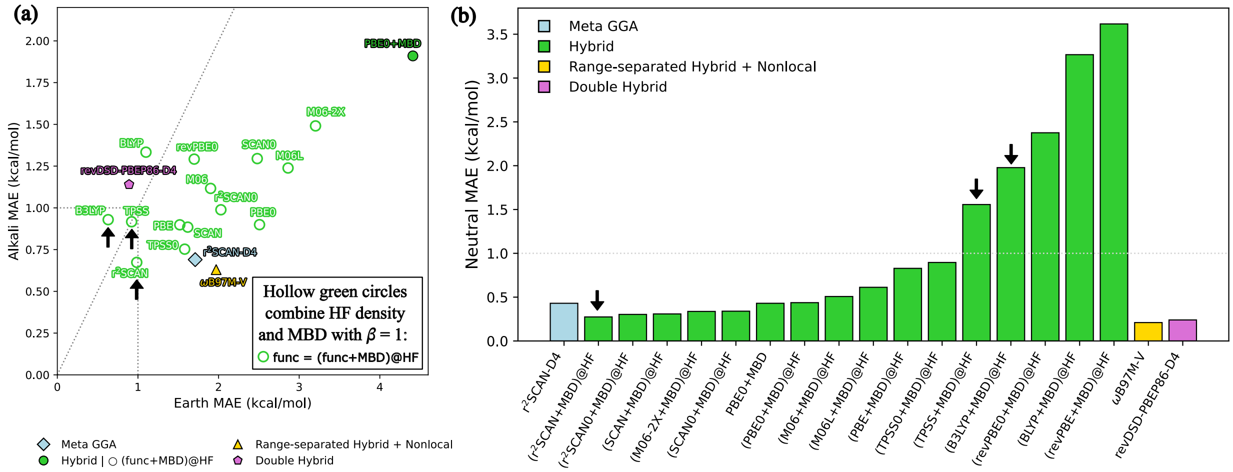

Figure S4: **MAEs on different DES15K subsets of methods at different levels of theory.** (a) Parity plots of the MAEs on the whole Earth subset from DES15K and the GA-selected Alkali subset (as shown in Fig. S2) of all tested methods, hollow circles indicate (DFT+MBD)@HF for different DFT functionals, along with MBD with β = 1. Arrows mark r^{2}SCAN, B3LYP, and TPSS functionals, highlighting those compared in panel (b). (b) MAEs of all tested methods on the GA-selected Neutral subset (see Fig. S2 for the underlying GA-subset selection.). Bars are colored by functional level of theory, matching panel (a). The computational cost increases across the method classes: meta-GGAs are cheapest; conventional hybrids (e.g., PBE0+MBD) and (DFT+MBD)@HF methods such as (r^{2}SCAN+MBD)@HF have comparable intermediate cost; range-separated hybrids with nonlocal correlation (e.g., ωB97M-V) are more costly than standard hybrids; and double hybrids are the most costly among the methods considered. **(r^{2}SCAN+MBD)@HF vs ωB97M-V wall times for representative system –** We benchmarked wall-clock times for a representative large charged NCI cluster from our MIPC set (PDB ID = 3Q6J: 108 atoms, 424 electrons) using ORCA 5.0.4 with 128 CPU cores and the def2-TZVPD basis with RIJCOSX approximation. The methods compared were ωB97M-V and (r^{2}SCAN+MBD)@HF, where r2SCAN energies are evaluated after a convergence of Hartree-Fock calculation; the MBD evaluation was negligible in cost relative to the SCF. On this system, wB97M-V required 13 min 50 s total wall time, while r2SCAN@HF required 7 min 55 s, i.e., r2SCAN@HF was ~1.75× faster under identical conditions. Calculations were performed on a dual-socket AMD system: 2× AMD EPYC 7742 64-Core Processor (128 hardware cores total, 1 thread per core, boost enabled), and 755 GiB RAM available on the node.

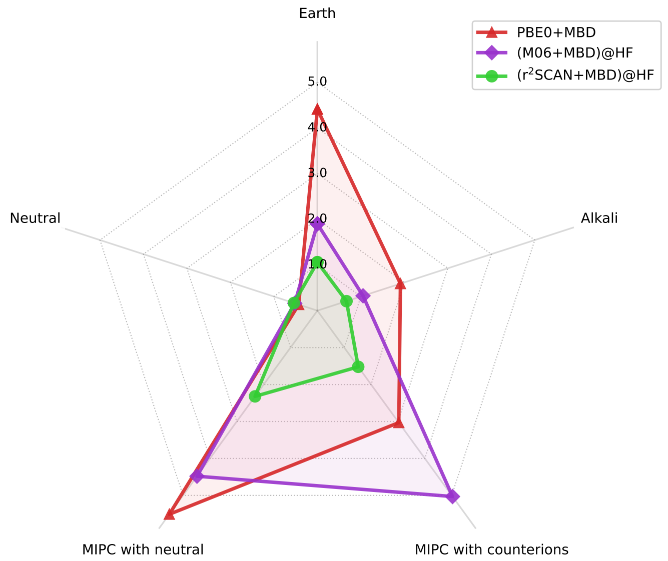

Figure S5: **Performance of three methods across chemical categories in DES15K and MIPC.** Radar plot of mean absolute errors (MAEs) in kcal/mol of interaction energies across 5 chemical categories of 3 methods. Earth denotes the whole Alkaline earth subset of DES15K; Alkali and Neutral denotes the GA-selected subset of DES15K (see Fig. S2 for details, GA denotes genetic algorithm); 'MIPC with neutral' denotes the MIPC subset of Fig. 3(b) containing neutral ligands; and 'MIPC with counterions' denotes the MIPC subset of Fig. 3(a) containing negatively charged ligands. The corresponding MAE values can be found in Tab. S7. For (M06+MBD)@HF, we report MAEs using the MBD range-separation parameter β as 1.0 (see Fig. S3).

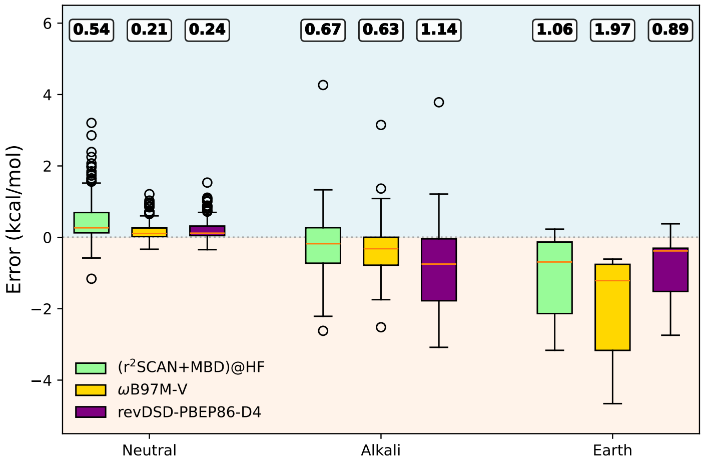

Figure S6: **Error distributions of three methods on GA-selected DES15K subsets.** Boxplots for (r^2SCAN+MBD)@HF, ωB97M-V and revDSD-PBEP86-D4 on the GA-selected Alkali and Neutral subsets of DES15K (see Fig. S2 for subset details, GA denotes genetic algorithm), as well as on the full Alkaline earth subset of DES15K. Mean absolute errors (MAEs) are shown above each box.

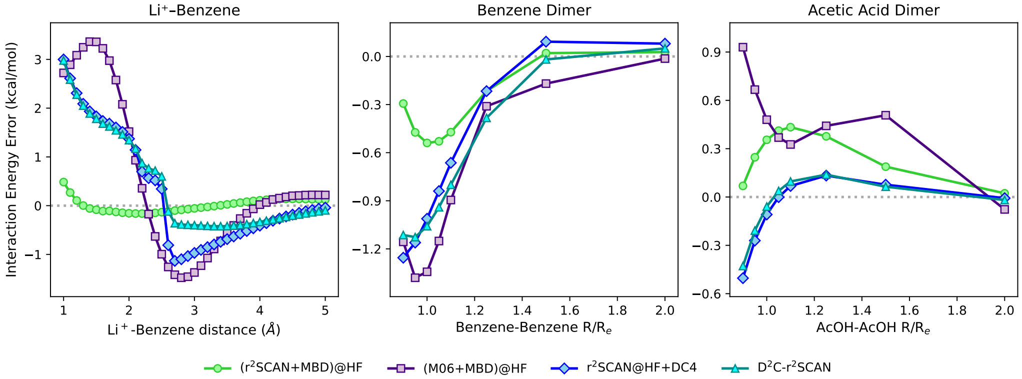

Figure S7: **Errors along dissociation curves for four methods.** Dissociation curves as in Fig. 5 showing errors of (r^2SCAN+MBD)@HF, (M06+MBD)@HF (with the empirical range-separation parameter in MBD set to $\beta = 1.0$), r^2SCAN@HF+DC4 (with D4 parameters taken from (*52*)) and D^2C-r^2SCAN, which is also r^2SCAN@HF+D4, but with the D4 parameters takens from (*53*).

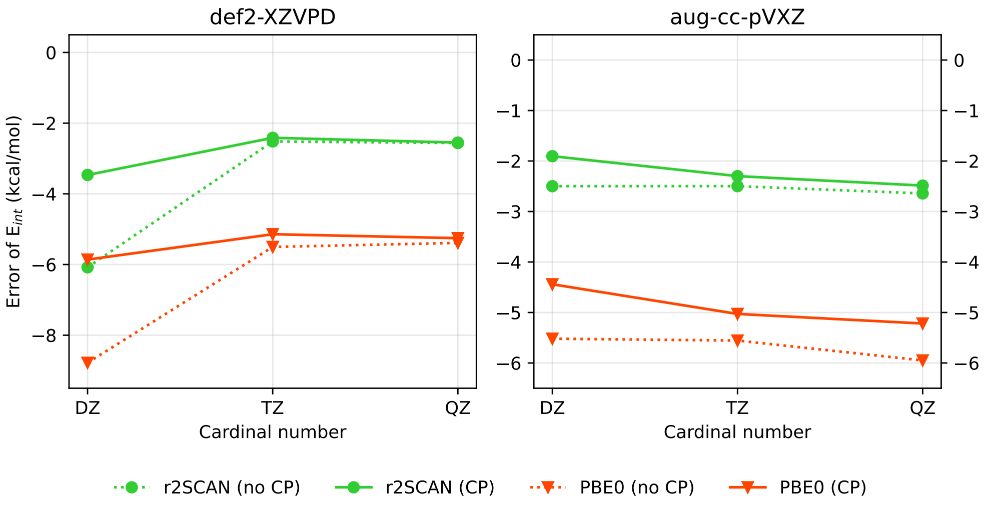

Figure S8: **Counterpoise correction and basis-set extrapolation for 2IJA (Na^+) interaction energies.** Errors in interaction energies for the complex were computed using different methods across the def2-XZVPD and aug-cc-pVXZ basis set series. Solid lines indicate counterpoise-corrected (CP) values, while dotted lines show uncorrected interaction energies, illustrating the magnitude of basis set superposition error (BSSE).

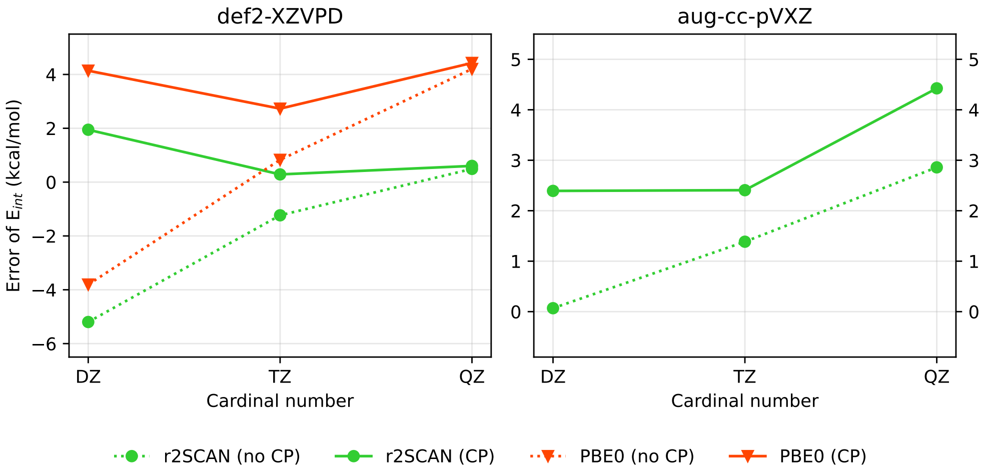

Figure S9: **Counterpoise correction and basis-set extrapolation for 2AP1 (Zn^{2+}) interaction energies.** Same as Fig. S8, but MBD@PBE0, i.e. MBD energies evaluated at PBE0 are not obtainable within any of the aug-cc basis set.

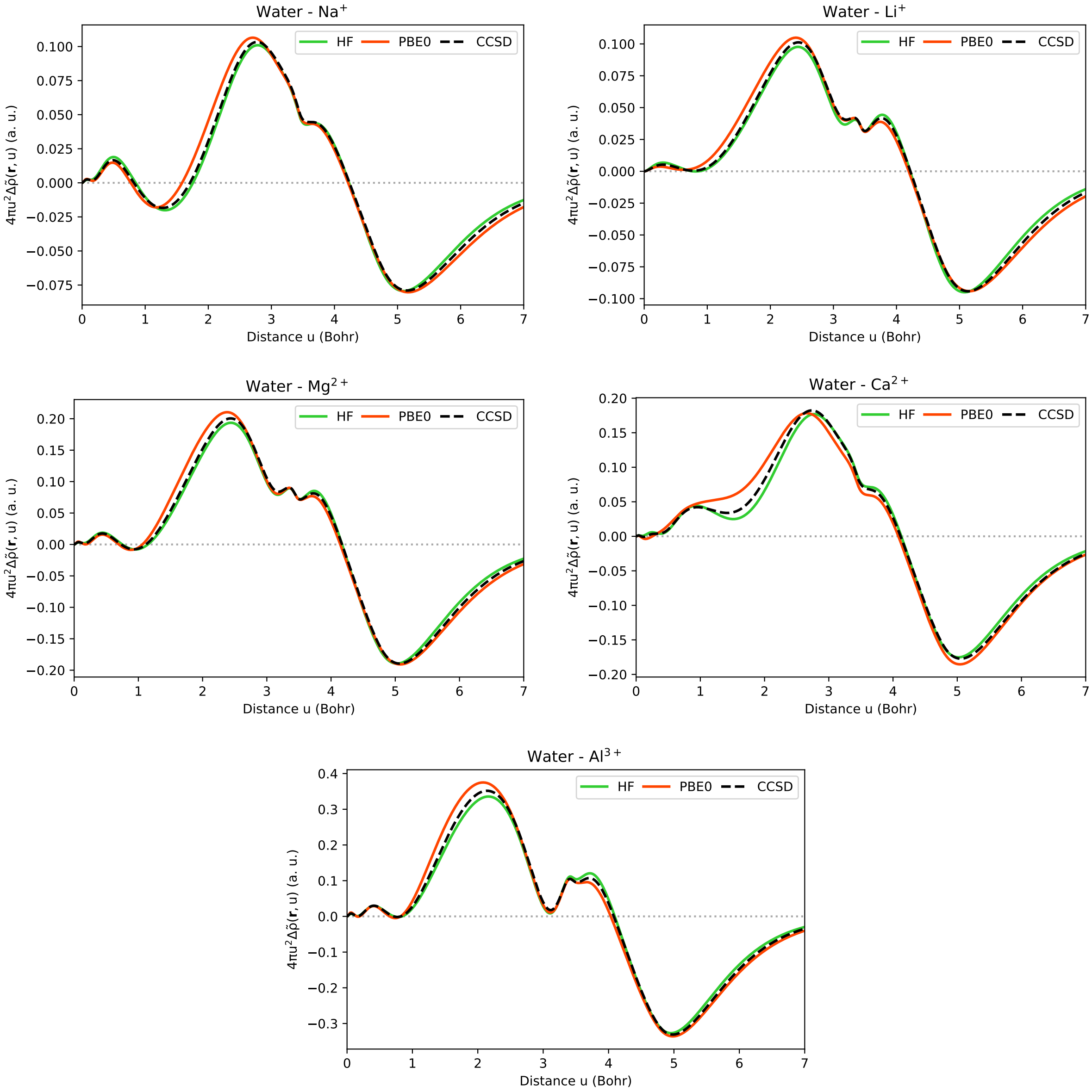

Figure S10: **Spherically averaged deformation densities for metal cation–water complexes.** Results were calculated using HF, PBE0 and CCSD methods, in which the origin of the spherical coordinate is placed at the position of metal cations.

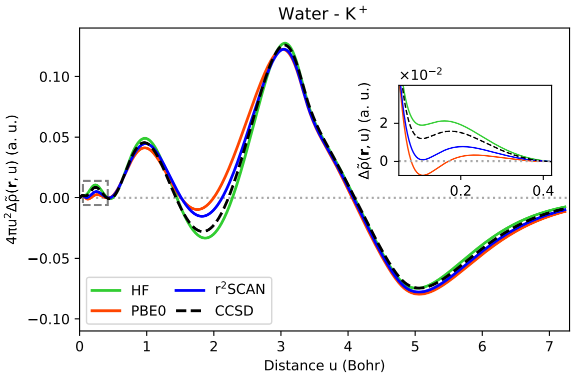

Figure S11: **Spherically averaged deformation densities for K$^+$–water complex.** Same as Fig. S10 but with additonal result calculated with r^{2}SCAN.

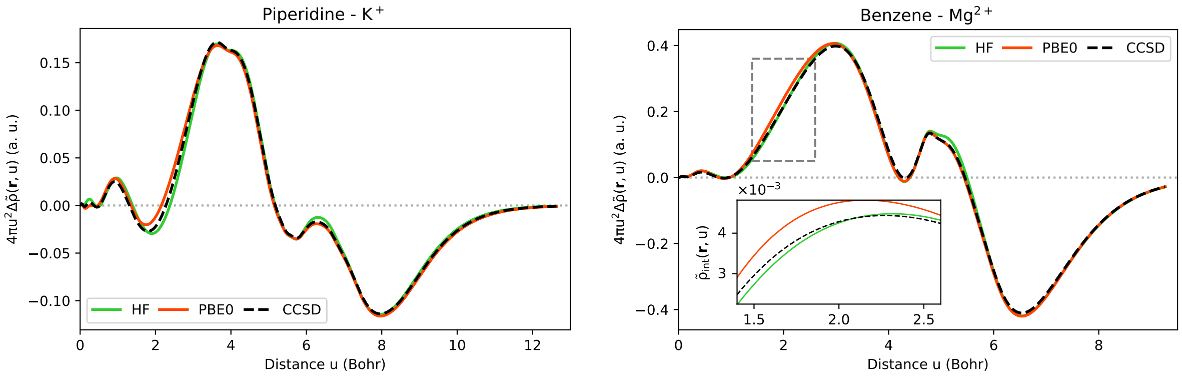

Figure S12: **Spherically averaged deformation densities for metal cation - molecule complexes.** Results of piperidine-K$^+$ (left), benzene-Mg^{2+} (right) complexes, calculated using HF, PBE0, and CCSD methods, in which the origin of the spherical coordinate is placed at the position of metal cations.

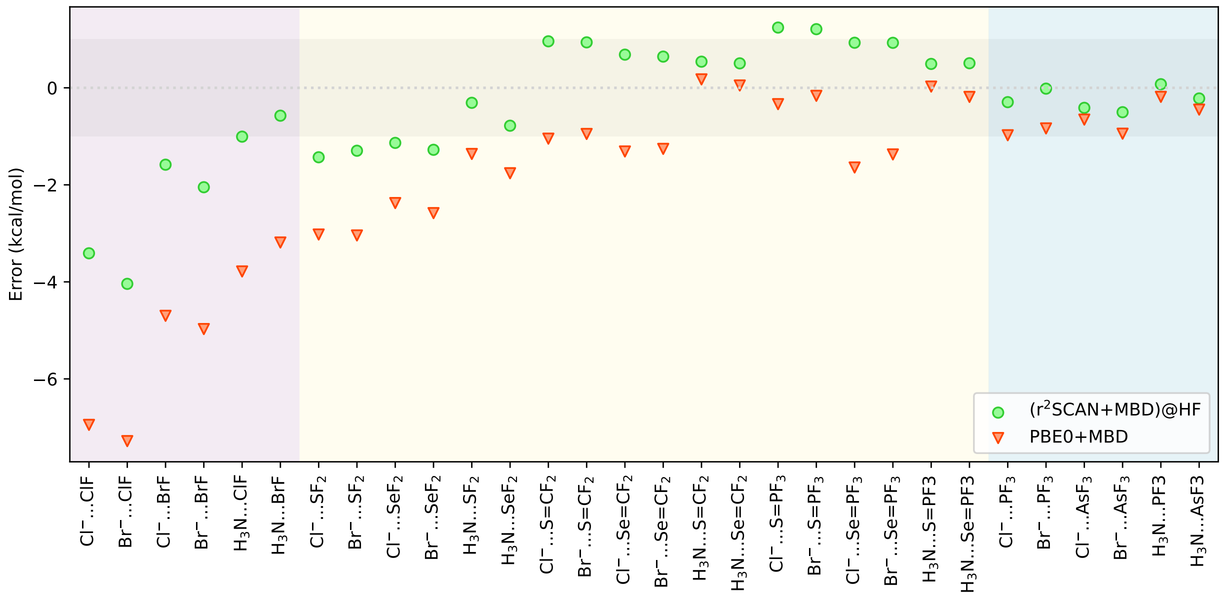

Figure S13: **Interaction energy errors of two methods for the B30 dataset.** Comparison of interaction energy errors (kcal/mol) of (r^2SCAN+MBD)@HF and PBE0+MBD for the B30 dataset, computed with the def2-QZVPPD basis set. the background color segments distinguish different types of NCIs (halogen, pnicogen, chalcogen) present in B30 set.

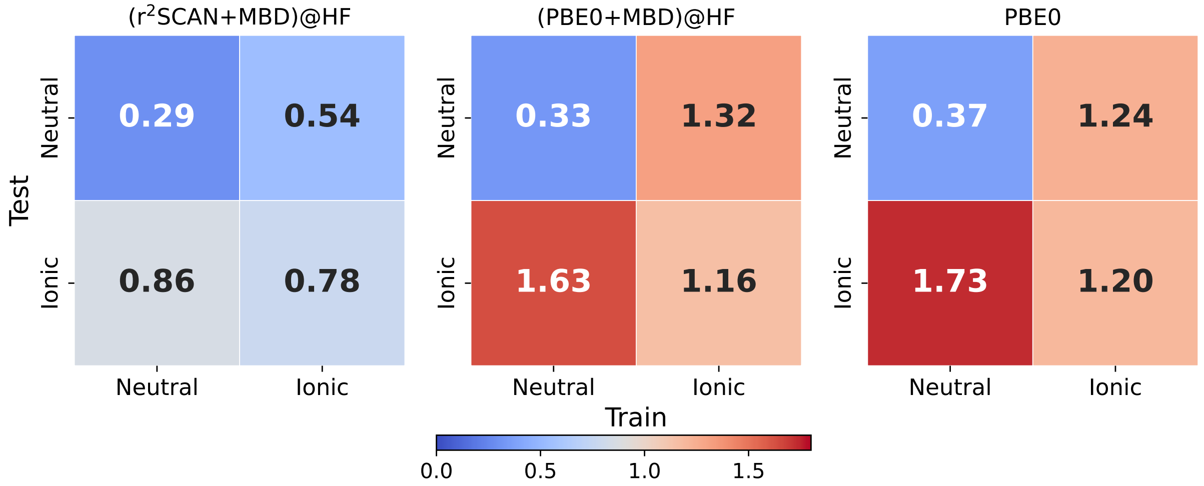

Figure S14: **Effect of MBD β choice on MAEs across datasets and methods.** Matrices showing MAEs (kcal/mol) for datasets in rows (metal, non-metal subsets of DES150 set consists of 50 metal, 50 non-metal ionic, and 50 neutral complexes selected from original DES15K) versus method with β values in MBD calculations trained on datasets in columns for three methods.

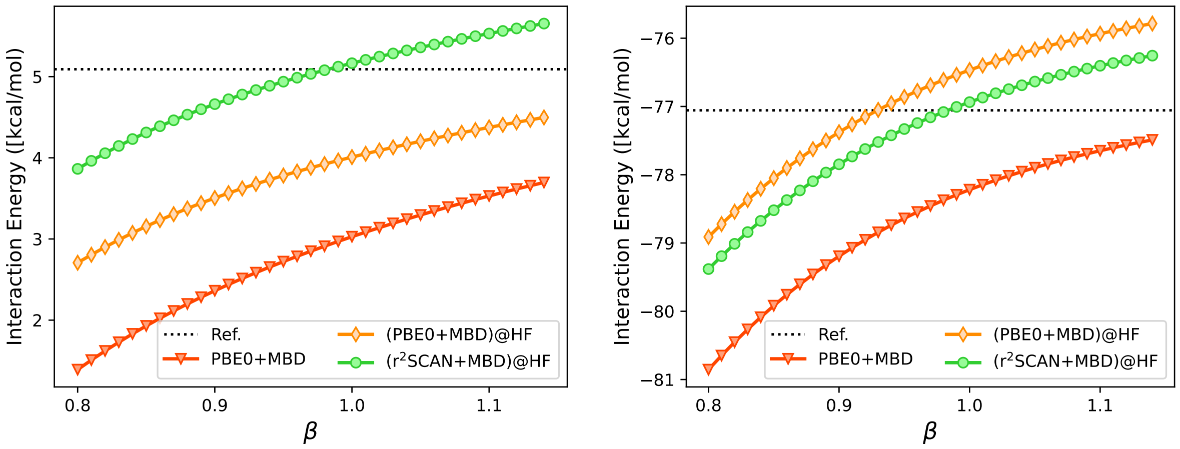

Figure S15: **Dependence of dispersion strength on parameter β in MBD.** Results shown for thiacyclohexane-K^+ (left) and Tetramethylammonium-Acetate (right).

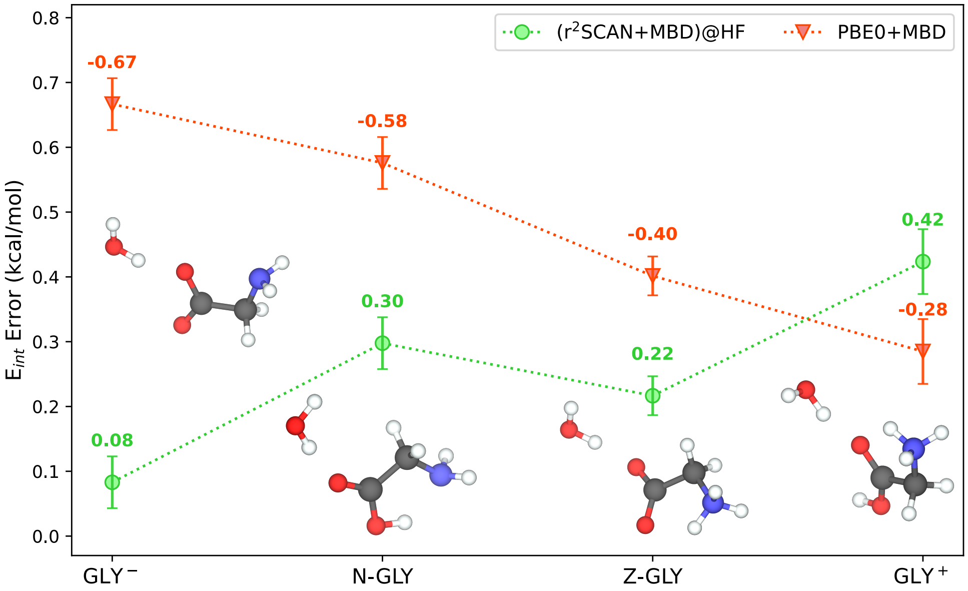

Figure S16: **Absolute interaction energy errors of (r^{2}SCAN+MBD)@HF and PBE0+MBD for four representative glycine-water complexes.** The complexes shown are GLY$^-$ (deprotonated neutral glycine), NGLY (neutral), ZGLY (zwitterionic), and GLY$^+$ (protonated zwitterion). Reference interaction energies were obtained using LNO-CCSD(T) as described in the Methods section, with the error bars on each points showing the uncertainty of the reference values, and the corresponding values are reported in Tab. S8. The Cartesian coordinates (xyz) of these complexes are provided in Supplementary Data S3.

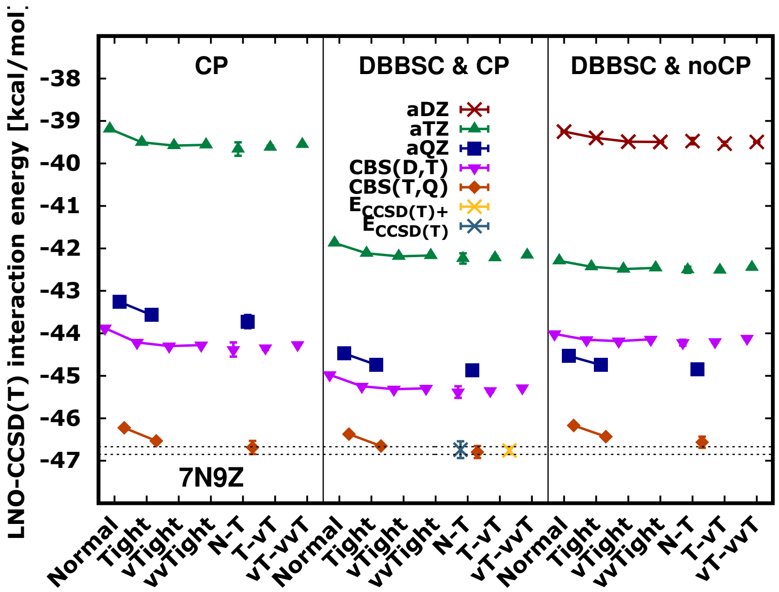

Figure S17: **Convergence of LNO-CCSD(T) correlation energies for the 7N9Z (Zn^{2+}) complex.** LNO-CCSD(T)/aug-cc-pV(X+d)Z (X = D, T, and Q) correlation energy contributions using various local correlation thresholds. HF/aug-cc-pV(Q+d)Z + CABS singles contribution: -291.23 kcal/mol.

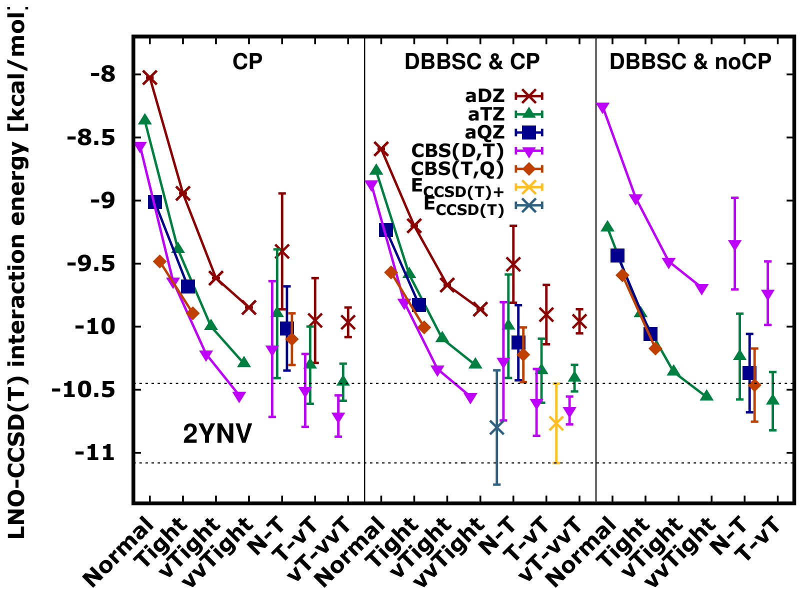

Figure S18: **Convergence of LNO-CCSD(T) correlation energies for the 2YNV (Mg^{2+}) complex.** LNO-CCSD(T)/aug-cc-pV(X+d)Z (X = D, T, and Q) correlation energy contributions using various local correlation thresholds. HF/aug-cc-pV(Q+d)Z + CABS singles contribution: -196.18 kcal/mol.

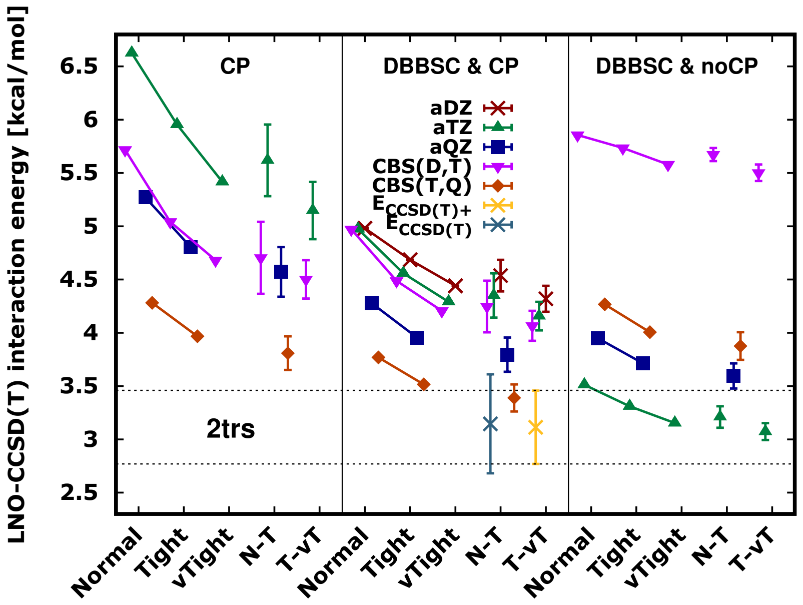

Figure S19: **Convergence of LNO-CCSD(T) correlation energies for the 2TRS (Na^+) complex.** LNO-CCSD(T)/aug-cc-pV(X+d)Z (X = D, T, and Q) correlation energy contributions using various local correlation thresholds. HF/aug-cc-pV(Q+d)Z + CABS singles contribution: -117.67 kcal/mol.

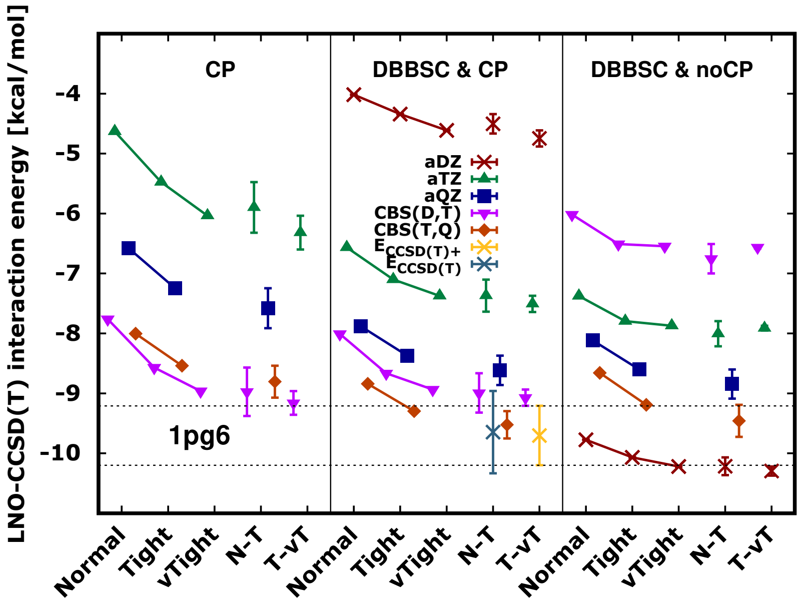

Figure S20: **Convergence of LNO-CCSD(T) correlation energies for the 1PG6 (Ca^{2+}) complex.** LNO-CCSD(T)/aug-cc-pV(X+d)Z (X = D, T, and Q) correlation energy contributions using various local correlation thresholds. HF/aug-cc-pV(Q+d)Z + CABS singles contribution: -240.71 kcal/mol.

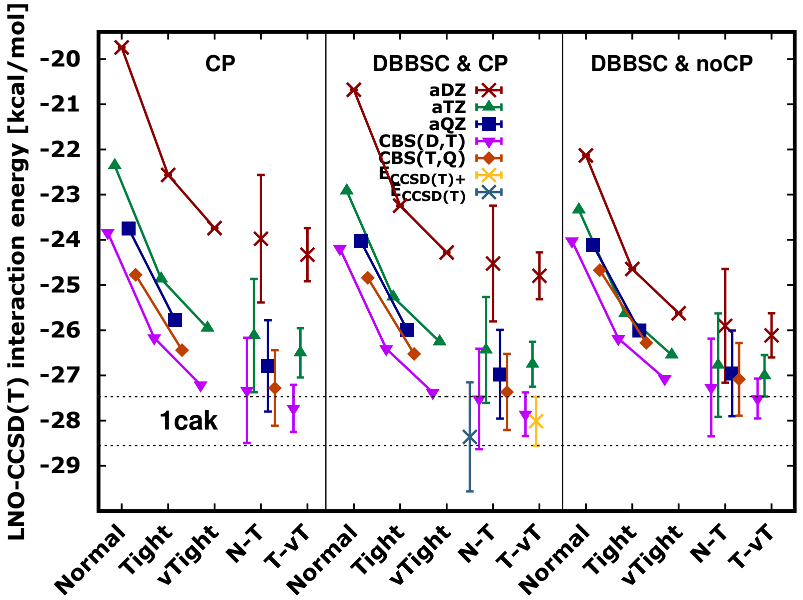

Figure S21: **Convergence of LNO-CCSD(T) correlation energies for the 1CAK (Zn^{2+}) complex.** LNO-CCSD(T)/aug-cc-pV(X+d)Z (X = D, T, and Q) correlation energy contributions using various local correlation thresholds. HF/aug-cc-pV(Q+d)Z + CABS singles contribution: -234.03 kcal/mol.

Table S1: **Size of each subset of DES15K in Figure 1.** The table reports, for each chemically defined subset, the number of complexes, the number of distinct monomers and dimers, and the mean span of the interaction energies over all separations of each distinct dimer.

Subset	No. Complexes	Unique Monomers	Unique Dimers	Mean span (kcal/mol)
Alkaline Earth	55	11	9	21.7
Alkali	903	133	217	27.3
Nonmetal Cation-Neutral	2060	132	479	27.1
Nonmetal Anion-Neutral	1273	130	290	26.3
Nonmetal Anion-Cation	97	14	26	22.1
Neutral	10263	135	2031	14.3

Table S2: **Source of the protein structures binding metal ions pertaining to the MIPC subset of Fig. 3(a).**

Cation	Source
Mg^{2+}	ccPDB 2.0 metal specific (*105*)
	highest resolution (1.00 Å) P-loop containing nucleoside triphosphate hydrolase structure containing Mg^{2+}+GTP (or analogue) (PDB ID 3X1X)
	highest resolution (0.95 Å) P-loop containing nucleoside triphosphate hydrolase structure containing Mg^{2+}+GDP (PDB ID 9IAY)
Zn^{2+}	EZAFF development set (*63*)
Ca^{2+}	ccPDB 2.0 metal specific (*105*)
K^{+}	AMOEBA development set (*106*)
Na^{+}	AMOEBA development set (*106*)

Table S3: **Residue profiles of the ion binding protein structures for the MIPC clusters used in Fig. 3(a).** 'Backbone CO' refers to the carbonyl group of the peptide bond of the protein backbone.

PDB ID	Ion	coordinating residues / moieties
2E85	Ca^{2+}	backbone CO; water ×3; ASP ×2
2YIK	Ca^{2+}	backbone CO; ASP ×3
2ZUY	Ca^{2+}	backbone CO; ASP ×3; GLU
3ETO	Ca^{2+}	backbone CO; ×2; ASP ×3; ASN
1JVJ	K^{+}	backbone CO; ×3; water; ASP
5AH1	K^{+}	water ×3; ASP ×2
6JXH	K^{+}	backbone CO; ×3; GLU ×2
1VM9	Mg^{2+}	water ×4; ASP ×2
1Y8A	Mg^{2+}	backbone CO; water ×3; ASP ×2
2G2S	Mg^{2+}	water ×5; ASP
3V5C	Mg^{2+}	water ×4; ASP; GLU
3X1X	Mg^{2+}	water ×3; SER; GTP(η^2)
9IAY	Mg^{2+}	water ×4; SER; GDP(η^1)
3VH9	Na^{+}	water ×4; GLU
5CVY	Na^{+}	water ×2; GLU; THR; -OH
6C9U	Na^{+}	backbone CO; ×3; GLU
1F57	Zn^{2+}	GLU; HIS ×2; $-S^-$
1H4N	Zn^{2+}	ASN; HIS ×2; $-NH_2$ $-O^-$
1Y9Q	Zn^{2+}	GLU; HIS ×2; TYR; $-COO^-$; $-NH_2$
2AP1	Zn^{2+}	CYS ×3; HIS

Table S4: **Residue profiles of the ion binding protein structures for the MIPC clusters used in Fig. 3(b).** 'Backbone CO' refers to the carbonyl group of the peptide bond of the protein backbone.

PDB ID	Ion	coordinating residues / moieties
3DXS	Li^+	water ×3; ASN
1L5B	Na^+	backbone CO ×2; water ×2; GLN ×2;
1VMF	Na^+	backbone CO ×3; water ×3;
2DSN	Na^+	water; SER; HIS; PHE(η^6)
2F53	Na^+	water ×2; GLN; THR
2ID4	Na^+	backbone CO ×3; water; SER
2IJA	Na^+	backbone CO; water ×3; TYR; THR
2Q6H	Na^+	backbone CO ×3; SER; THR
2RKA	Na^+	backbone CO ×2; water ×2
2TRS	Na^+	backbone CO ×3; water ×2
7O20	Na^+	backbone CO ×2; water; SER; THR
2YNV	Mg^{2+}	backbone CO; water; SER; HIS
3Q6J	Mg^{2+}	backbone CO ×3; water; SER
4YN0	Mg^{2+}	backbone CO ×3; water; SER
1PG6	Ca^{2+}	backbone CO ×2; water; ASN; THR
1POD	Ca^{2+}	backbone CO ×4; water
2C1U	Ca^{2+}	backbone CO ×2; water ×4; ASN
2P5W	Ca^{2+}	backbone CO ×2; water; ASN
3HQ8	Ca^{2+}	backbone CO ×2; water ×2; ASN
3L4M	Ca^{2+}	backbone CO ×2; water ×4; ASN
1CAK	Zn^{2+}	backbone CO ×3; water ×2
1HTV	Zn^{2+}	water ×3; HIS ×3
1M65	Zn^{2+}	water; HIS ×3
4JEB	Zn^{2+}	water; HIS ×3
7N9Z	Zn^{2+}	MET ×4
8OOD*	Mg^{2+}	backbone CO ×3; water
(due to error, heteroatom chains not considered and thus excluded)		

Table S5: **Interaction energies of MIPC with negatively charged ligands from Fig. 3(a).** Results are in kcal/mol, calculated using the (r^2SCAN+MBD)@HF, PBE0+MBD methods, compared to reference values obtained using LNO-CCSD(T) with its uncertainty, as described in Methods section. The Cartesian coordinates (xyz) can be found in Supplementary Data S2.

PDB ID	ion type	(r^2SCAN+MBD)@HF	PBE0+MBD	LNO-CCSD(T)	Uncertainty
2E85	Ca^{2+}	-612.40	-616.31	-615.21	0.30
2YIK	Ca^{2+}	-686.99	-690.96	-687.92	0.16
2ZUY	Ca^{2+}	-864.77	-868.39	-866.26	0.30
3ETO	Ca^{2+}	-739.81	-744.34	-741.46	0.52
1JVJ	K^{+}	-93.37	-97.67	-90.80	0.10
5AH1	K^{+}	-220.08	–	-220.14	0.24
6JXH	K^{+}	-301.56	-307.19	-297.34	0.37
1VM9	Mg^{2+}	-692.36	-693.97	-692.67	0.22
1Y8A	Mg^{2+}	-700.35	-702.66	-699.81	0.30
2G2S	Mg^{2+}	-505.89	-507.55	-506.31	0.24
3V5C	Mg^{2+}	-652.35	-654.61	-652.34	0.19
3X1X	Mg^{2+}	-941.03	-938.92	-940.87	0.21
9IAY	Mg^{2+}	-815.74	-815.10	-815.23	0.20
3VH9	Na^{+}	-141.65	-144.06	-139.32	0.06
5CVY	Na^{+}	-138.22	-141.56	-135.60	0.08
6C9U	Na^{+}	-194.66	-198.28	-191.53	0.11
1F57	Zn^{2+}	-743.13	-740.17	-741.82	0.12
1H4N	Zn^{2+}	-596.20	-595.00	-594.61	0.19
1Y9Q	Zn^{2+}	-763.33	-759.55	-761.13	0.03
2AP1	Zn^{2+}	-901.53	-899.47	-900.30	1.40
MAE		1.51	3.03		

Table S6: **Interaction energies of MIPC with neutral ligands Fig. 3(b).** Results are in kcal/mol, calculated using the (r^2SCAN+MBD)@HF, PBE0+MBD methods, compared to reference values obtained using LNO-CCSD(T) with its uncertainty, as described in Methods section. The Cartesian coordinates (xyz) can be found in Supplementary Data S2.

PDB ID	ion type	(r^2SCAN+MBD)@HF	PBE0+MBD	LNO-CCSD(T)	Uncertainty
1POD	Ca^{2+}	-278.27	-283.37	-278.61	0.61
1PG6	Ca^{2+}	-251.13	-256.86	-250.41	0.50
2P5W	Ca^{2+}	-228.51	-233.02	-229.12	0.38
2C1U	Ca^{2+}	-325.49	-328.76	-326.48	0.70
3L4M	Ca^{2+}	-319.37	-322.56	-320.30	0.64
3HQ8	Ca^{2+}	-287.55	-292.49	-289.85	0.61
3DXS	Li^{+}	-112.75	-116.53	-113.00	0.18
2YNV	Mg^{2+}	-209.21	-216.09	-206.95	0.32
3Q6J	Mg^{2+}	-345.07	-349.30	-342.22	0.75
4YN0	Mg^{2+}	-334.93	-339.04	-333.60	0.66
1L5B	Na^{+}	-118.51	-122.10	-114.60	0.27
1VMF	Na^{+}	-148.97	-152.01	-144.30	0.33
2DSN	Na^{+}	-56.93	-60.75	-53.60	0.08
2F53	Na^{+}	-73.99	-77.71	-70.84	0.18
2ID4	Na^{+}	-117.75	-121.39	-114.62	0.27
2IJA	Na^{+}	-89.53	-92.51	-87.01	0.13
2Q6H	Na^{+}	-126.34	-130.25	-122.82	0.37
2RKA	Na^{+}	-88.39	-91.44	-85.74	0.15
2TRS	Na^{+}	-120.03	-123.94	-114.56	0.34
7O20	Na^{+}	-118.53	-122.42	-115.05	0.23
1M65	Zn^{2+}	-419.31	-417.86	-418.98	0.27
1CAK	Zn^{2+}	-265.74	-273.85	-262.04	0.54
1HTV	Zn^{2+}	-408.65	-408.18	-406.78	0.46
4JEB	Zn^{2+}	-394.84	-394.82	-393.58	0.48
7N9Z	Zn^{2+}	-340.35	-341.28	-337.99	0.09
MAE		2.32	5.51		
8OOD*	Mg^{2+}	-350.12	-354.18	-350.14	0.51

(Due to error, heteroatom chains not considered for 8OOD and thus excluded.)

Table S7: **Mean absolute errors (kcal/mol) for different methods across various NCI sets.** "Alkaline earth" refers to the 55 earth-alkaline complexes in the original DES15K Metal category."Alkali" corresponds to the 97 alkali complexes in the GA-selected Metal subset from DES15K. "Neutral" corresponds to the 200 complexes in the GA-selected Neutral subset from DES15K (see Fig. S2 for details, GA denotes genetic algorithm). For (M06+MBD)@HF, we report MAEs using the MBD range-separation parameter β as 1.0 (see Fig. S3).

Method	Alkaline earth (DES15K)	Alkali (GA)	Neutral (GA)
(r^2SCAN+MBD)@HF	1.06	0.67	0.54
PBE0+MBD	4.41	1.91	0.43
(M06+MBD)@HF	1.90	1.12	0.52
ωB97M-V	1.97	0.63	0.21
revDSD-PBEP86-D4	0.89	1.14	0.24

Table S8: **Interaction energies for representative glycine-water clusters in Fig. S16.** Values are in kcal/mol, calculated using the (r^2SCAN+MBD)@HF, PBE0+MBD methods, compared to reference values obtained using LNO-CCSD(T) with its uncertainty, as described in Methods section. The systems selected are GLY^- (deprotonated neutral glycine), NGLY (neutral), ZGLY (zwitterionic), and GLY^+ (protonated zwitterion), and their Cartesian coordinates (xyz) can be found in Supplementary Data S3.

System	(r^2SCAN+MBD)@HF	PBE0+MBD	LNO-CCSD(T)	Uncertainty
GLY^-	-14.37	-15.12	-14.45	0.04
N-GLY	-1.45	-2.33	-1.75	0.04
Z-GLY	-4.8	-5.42	-5.02	0.03
GLY^+	4.68	3.98	4.26	0.05

Table S9: **Interaction energies for glycine–water clusters.** (continued on the next page) Cluster geometries are taken from Ref. (*78*), comprising neutral glycine (NGLY), zwitterionic glycine (ZGLY), and proton transfer transition state (TS) complexes, and the "Frame" index follows the same numbering as in Ref. (*78*), so that each frame corresponds to the identical glycine–water geometry used in the original work. TS complexes correspond to proton transfer process between NGLY and ZGLY. Results are in kcal/mol, calculated using the (r^2SCAN+MBD)@HF, PBE0+MBD methods, compared to reference interaction energies recomputed using LNO-CCSD(T) with its uncertainty, as described in Methods section.

System	No. H_2O	Frame	(r^2SCAN+MBD)@HF	PBE0+MBD	LNO-CCSD(T)	Uncertainty
NGLY	1	0	-1.18	-2.16	-1.46	0.05
NGLY	1	6	-1.82	-2.45	-2.09	0.03
NGLY	1	7	2.77	1.93	2.15	0.03
NGLY	1	8	-2.73	-3.41	-3.06	0.03
NGLY	1	9	-2.27	-3.01	-2.54	0.04
NGLY	1	10	1.82	1.01	1.25	0.03
NGLY	1	11	-1.45	-2.33	-1.75	0.04
NGLY	1	13	0.26	-0.33	-0.21	0.03
NGLY	1	14	-3.68	-4.28	-4.0	0.04
NGLY	1	17	-1.82	-2.51	-2.09	0.04
NGLY	3	1	-3.09	-4.87	-4.12	0.08
NGLY	3	7	-3.21	-4.94	-4.55	0.12
NGLY	3	8	-4.58	-6.41	-5.75	0.09
NGLY	3	10	-5.09	-7.05	-6.1	0.09
NGLY	3	13	-5.73	-7.18	-6.83	0.07
NGLY	5	1	-11.15	-13.97	-12.8	0.13
NGLY	5	7	-12.67	-14.59	-14.4	0.12
NGLY	5	8	-8.97	-11.2	-10.6	0.14
NGLY	10	16	-19.45	-22.08	-21.4	0.21
MAE NGLY			0.86	0.50		
TS	1	0	-2.81	-3.44	-3.24	0.04
TS	1	3	-3.21	-3.92	-3.49	0.04
TS	1	7	-3.4	-4.13	-3.78	0.05
TS	1	8	-1.95	-2.55	-2.4	0.03
TS	1	9	-1.97	-2.71	-2.48	0.04

System	No. H_2O	Frame	(r^2SCAN+MBD)@HF	PBE0+MBD	LNO-CCSD(T)	Uncertainty
TS	1	10	-2.1	-2.84	-2.42	0.05
TS	1	11	-2.77	-3.4	-3.23	0.03
TS	1	13	-0.31	-1.2	-0.6	0.04
TS	1	16	-4.66	-5.24	-4.97	0.03
TS	1	18	-4.6	-5.29	-4.95	0.04
TS	3	0	-8.73	-10.02	-9.72	0.11
TS	3	3	-9.49	-10.98	-10.52	0.1
TS	3	10	-7.33	-8.78	-8.44	0.09
TS	3	13	-9.58	-11.55	-10.81	0.1
TS	3	14	-10.23	-12.3	-11.37	0.11
TS	5	10	-16.46	-19.07	-18.26	0.15
TS	5	14	-22.58	-25.8	-24.73	0.19
TS	5	18	-13.45	-15.38	-15.21	0.11
TS	10	6	-24.02	-27.26	-26.4	0.18
MAE TS			0.91	0.47		
ZGLY	1	1	-6.0	-6.68	-6.33	0.04
ZGLY	1	3	-11.2	-11.54	-11.47	0.05
ZGLY	1	4	-2.0	-2.43	-2.34	0.05
ZGLY	1	5	-6.25	-6.74	-6.58	0.04
ZGLY	1	7	-4.8	-5.42	-5.02	0.03
ZGLY	1	8	-4.3	-4.81	-4.65	0.04
ZGLY	1	9	-5.0	-5.63	-5.34	0.05
ZGLY	1	13	-7.11	-7.63	-7.5	0.04
ZGLY	1	16	-8.58	-9.28	-8.86	0.06
ZGLY	1	17	-4.97	-5.63	-5.42	0.04
ZGLY	3	1	-20.7	-22.2	-21.75	0.11
ZGLY	3	2	-25.82	-27.29	-26.85	0.12
ZGLY	3	5	-18.04	-19.64	-19.08	0.13
ZGLY	3	7	-14.93	-16.13	-15.88	0.12
ZGLY	3	12	-15.38	-17.09	-16.21	0.13
ZGLY	5	1	-30.15	-31.88	-31.88	0.18
ZGLY	5	7	-27.72	-28.98	-29.19	0.16
ZGLY	5	12	-22.64	-24.88	-24.25	0.18
ZGLY	10	3	-57.95	-60.46	-60.67	0.23
MAE ZGLY			0.83	0.31		
MAE ALL			0.87	0.42		

Table S10: **Interaction energies for methods on B30 dataset.** Results are in kcal/mol, calculated using the (r^2SCAN+MBD)@HF, PBE0+MBD methods with def2-QZVPPD basis set, compared to reference values from Ref. (*57*).

Complex	Ref	(r^2SCAN+MBD)@HF	PBE0+MBD
Cl^-...ClF	-43.85	-47.26	-50.80
Br^-...ClF	-42.59	-46.63	-49.87
Cl^-...BrF	-46.38	-47.96	-51.08
Br^-...BrF	-43.67	-45.72	-48.64
H_3N...ClF	-12.07	-13.08	-15.86
H_3N...BrF	-16.03	-16.60	-19.22
MAE (Halogen)		2.11	5.15
Cl^-...SF_2	-31.49	-32.92	-34.51
Br^-...SF_2	-25.81	-27.11	-28.85
Cl^-...SeF_2	-40.63	-41.76	-43.01
Br^-...SeF_2	-35.28	-36.56	-37.86
H_3N...SF_2	-8.00	-8.31	-9.36
H_3N...SeF_2	-12.89	-13.67	-14.65
Cl^-...S=CF_2	-9.52	-8.56	-10.57
Br^-...S=CF_2	-7.79	-6.85	-8.74
Cl^-...Se=CF_2	-13.52	-12.84	-14.83
Br^-...Se=CF_2	-11.15	-10.51	-12.41
H_3N...S=CF_2	-1.68	-1.14	-1.51
H_3N...Se=CF_2	-2.55	-2.05	-2.50
Cl^-...S=PF_3	-8.41	-7.17	-8.75
Br^-...S=PF_3	-6.75	-5.54	-6.92
Cl^-...Se=PF_3	-15.26	-14.33	-16.90
Br^-...Se=PF_3	-12.31	-11.38	-13.68
H_3N...S=PF3	-1.42	-0.93	-1.39
H_3N...Se=PF3	-2.64	-2.13	-2.83
MAE (Chalcogen)		0.88	1.26
Cl^-...PF_3	-21.33	-21.63	-22.31
Br^-...PF_3	-15.73	-15.75	-16.57
Cl^-...AsF_3	-34.06	-34.47	-34.72
Br^-...AsF_3	-27.31	-27.81	-28.26
H_3N...PF3	-4.86	-4.79	-5.05
H_3N...AsF3	-9.18	-9.40	-9.63
MAE (Pnictogen)		0.25	0.68
MAE (Anion-Neutral)		1.25	2.32
MAE (Overall)		1.00	1.92

Table S11: **Mean absolute errors (MAEs) and WTMAD-2 (kcal/mol) for various methods across subsets of the GMTKN55 dataset.** (continued on the next page)

Set	B3LYP-D4	PBE0-D4	SCAN-D4	r^2SCAN-D4	r^2SCAN@HF-DC4	(r^2SCAN+MBD)@HF
Basic						
W4-11	3.16	3.52	3.46	3.86	6.48	6.72
G21EA	2.31	2.85	3.96	3.83	4.55	4.59
G21IP	3.74	3.55	4.84	4.66	4.54	4.56
DIPCS10	4.57	2.93	4.97	5.14	4.35	4.40
PA26	1.97	2.29	2.95	2.40	1.80	1.88
SIE4x4	17.72	14.15	17.76	18.02	12.24	12.21
ALKBDE10	4.33	5.54	5.11	5.01	4.68	4.67
YBDE18	4.72	0.96	3.31	3.37	3.60	3.65
AL2X6	3.23	1.32	1.84	1.58	1.09	1.10
HEAVYSB11	3.29	1.40	2.10	3.23	4.62	4.90
NBPRC	2.00	3.07	2.34	1.52	1.05	0.96
ALK8	3.48	1.74	2.42	2.23	1.33	5.80
RC21	2.43	5.46	5.77	4.98	2.44	2.56
G2RC	2.57	6.62	6.21	5.55	4.50	4.40
BH76RC	1.95	2.17	3.11	2.98	2.56	2.56
FH51	2.51	2.71	2.62	2.16	1.68	1.62
TAUT15	1.14	1.13	1.76	1.57	1.08	1.06
DC13	9.46	8.30	6.93	7.72	8.09	8.31
Large						
MB16-43	28.47	15.73	16.52	14.12	9.96	9.65
DARC	7.62	3.94	2.16	2.70	2.09	2.40
RSE43	1.82	1.50	1.34	1.54	0.95	0.95
BSR36	2.51	2.77	1.66	0.48	0.33	0.96
CDIE20	1.08	1.28	1.47	1.61	1.14	1.15
ISO34	1.74	1.43	1.32	1.29	1.37	1.43
PArel	1.14	1.19	1.50	1.54	1.14	1.14
C60ISO	2.63	2.27	6.13	5.57	3.74	3.58
ISOL24	5.38	2.11	3.49	4.10	3.03	3.10
Barrier						
BH76	5.04	4.18	7.47	6.99	2.90	2.87
BHPERI	1.13	3.26	5.16	4.65	5.68	5.07
BHDIV10	3.23	4.80	6.55	6.11	3.77	3.60
INV24	1.02	1.13	1.09	1.14	1.33	1.32
BHROT27	0.42	0.58	0.85	0.76	0.63	0.63
PX13	4.17	6.39	8.10	8.81	5.00	4.78
WCPT18	2.06	4.16	6.00	5.99	3.13	2.92

Set	B3LYP-D4	PBE0-D4	SCAN-D4	r^2SCAN-D4	r^2SCAN@HF-DC4	(r^2SCAN+MBD)@HF
Inter NCI						
RG18	0.16	0.07	0.17	0.16	0.12	0.12
ADIM6	0.24	0.16	0.46	0.34	0.31	0.44
S22	0.42	0.38	0.36	0.24	0.44	0.34
S66	0.30	0.31	0.35	0.26	0.29	0.28
HEAVY28	0.21	0.25	0.29	0.30	0.45	0.58
WATER27	3.02	4.91	8.46	6.30	0.95	1.48
CARBHB12	0.64	1.27	1.23	1.06	0.57	0.55
PNICO23	0.31	0.82	0.94	0.76	0.26	0.36
HAL59	0.54	0.60	1.01	0.80	0.53	0.66
AHB21	0.41	1.29	1.61	1.27	0.65	0.60
CHB6	0.96	0.98	0.44	0.52	0.56	0.70
IL16	0.33	0.47	0.88	0.64	0.47	1.05
Intra NCI						
IDISP	5.87	3.59	7.13	7.21	1.63	2.45
ICONF	0.28	0.27	0.30	0.29	0.25	0.27
ACONF	0.06	0.06	0.22	0.18	0.17	0.27
Amino20x4	0.20	0.25	0.23	0.19	0.27	0.32
PCONF21	0.36	0.75	0.40	0.42	0.48	0.41
MCONF	0.26	0.27	0.43	0.45	0.25	0.16
SCONF	0.31	0.28	0.56	0.51	0.22	0.39
BUT14DIOL	0.46	0.26	0.29	0.23	0.16	0.29
UPU23	0.58	0.52	0.39	0.41	0.39	0.40
WTMAD-2						
Basic	4.15	4.31	5.21	4.91	4.08	4.14
Large	9.87	8.21	8.16	8.15	5.92	6.33
Barrier	8.18	8.83	14.36	13.55	7.91	7.58
Inter NCI	4.96	5.75	8.04	6.76	5.56	6.52
Intra NCI	6.18	6.21	6.45	5.92	5.11	6.06
Overall	6.15	6.18	7.68	7.12	5.37	5.79

Caption for Data S1. Genetic-algorithm-selected DES15K dimers. The file contains dimers drawn from the DES15K benchmark, with basic monomer descriptors, links back to the original DES15K geometries, and CCSD(T)/CBS reference interaction energy plus two DFT interaction energies ((r^2SCAN+MBD)@HF and PBE0+MBD), all in kcal/mol.

Columns are: `smiles0` and `smiles1`, SMILES strings for monomer 0 and 1; `charge0` and `charge1`, formal integer charges of each monomer; `natoms0` and `natoms1`, total atom counts in each monomer. `system_id` is the DES15K system identifier for the dimer, while `group_orig` records the origin of the geometry in DES15K (e.g. `qm_opt_dimer`, `md_dimer`); `group_id` labels a specific scan/trajectory within a system, and `k_index` is the integer index along the DES15K distance scan for that group; `geom_id` is the unique DES15K geometry identifier that can be used to retrieve coordinates. `subset` gives the type of NCIs also as subset name (e.g. `earth`). `CCSDt_CBS` is the CCSD(T)/CBS dimer interaction energy, while `(r2SCAN+MBD)@HF` and `PBE0+MBD` are corresponding interaction energies from the indicated DFT+MBD methods; all three energy columns are defined as $E_{\text{int}} = E_{\text{dimer}} - E_{\text{mon0}} - E_{\text{mon1}}$ in kcal/mol, with negative values indicating net attraction.

Caption for Data S2. XYZ geometries of all MIPC complexes. This tar archive contains the three-dimensional Cartesian coordinates (XYZ format) for every complex in the MIPC dataset, with each file named by its corresponding PDB ID to allow direct matching to the entries reported in the Fig. 3, Tab. S5 and Tab. S6.

Caption for Data S3. XYZ geometries of representative glycine-water complexes. This tar archive contains the three-dimensional Cartesian coordinates (XYZ format) for all four glycine-water complexes in the Fig. S16 and Tab. S8.

Caption for Data S4. Single point electronic energies of all molecules in this work. This spreadsheet contains eight tabs. `MIPC_neutral_LNOCC` and `MIPC_charged_LNOCC` report the raw energy components (kcal/mol) entering Eq. 1 for the neutral and charged MIPC sets, respectively. `MIPC_neutral_DFT` and `MIPC_charged_DFT` list single-point total energies (Hartree) for (r^2SCAN+MBD)@HF and PBE0+MBD for the corresponding neutral and charged MIPC molecules. For the GA subset, `GA_subset_Complex` provides complex energies (Hartree) for the

tested (func+MBD)@HF methods, and `GA_subset_Monomer` provides the associated monomer energies. For DES15k, `DES15k_Complex` reports complex energies (Hartree) at (r^2SCAN+MBD)@HF and PBE0+MBD, while `DES15k_Monomer` reports energies for the 159 unique monomers appearing in those complexes.

REFERENCES

1. V. A. Adhav, K. Saikrishnan, The realm of unconventional noncovalent interactions in proteins: Their significance in structure and function. *ACS Omega* **8**, 22268–22284 (2023).

2. R. W. Newberry, R. T. Raines, Secondary forces in protein folding. *ACS Chem. Biol.* **14**, 1677–1686 (2019).

3. M. Rossi, A. Tkatchenko, S. B. Rempe, S. Varma, Role of methyl-induced polarization in ion binding. *Proc. Natl. Acad. Sci. U.S.A.* **110**, 12978–12983 (2013).

4. S. Yamada, Cation-π interactions in organic synthesis. *Chem. Rev.* **118**, 11353–11432 (2018).

5. G. Maurin, C. Serre, A. Cooper, G. Férey, The new age of MOFs and of their porous-related solids. *Chem. Soc. Rev.* **46**, 3104–3107 (2017).

6. J.-X. Huang, G. Csányi, J.-B. Zhao, J. Cheng, V. L. Deringer, First-principles study of alkali-metal intercalation in disordered carbon anode materials. *J. Mater. Chem. A* **7**, 19070–19080 (2019).

7. K. Fumino, S. Reimann, R. Ludwig, Probing molecular interaction in ionic liquids by low frequency spectroscopy: Coulomb energy, hydrogen bonding and dispersion forces. *Phys. Chem. Chem. Phys.* **16**, 21903–21929 (2014).

8. B. Huang, S. Muy, S. Feng, Y. Katayama, Y. C. Lu, G. Chen, Y. Shao-Horn, Non-covalent interactions in electrochemical reactions and implications in clean energy applications. *Phys. Chem. Chem. Phys.* **20**, 15680–15686 (2018).

9. J. Rezac, P. Hobza, Describing noncovalent interactions beyond the common approximations: How accurate is the "gold standard," CCSD(T) at the complete basis set limit? *J. Chem. Theory Comput.* **9**, 2151–2155 (2013).

10. W. M. Foulkes, L. Mitas, R. Needs, G. Rajagopal, Quantum Monte Carlo simulations of solids. *Rev. Mod. Phys.* **73**, 33–83 (2001).

11. C. Riplinger, F. Neese, An efficient and near linear scaling pair natural orbital based local coupled cluster method. *J. Chem. Phys.* **138**, 034106 (2013).

12. P. R. Nagy, M. Kállay, Approaching the basis set limit of CCSD(T) energies for large molecules with local natural orbital coupled-cluster methods. *J. Chem. Theory Comput.* **15**, 5275–5298 (2019).

13. P. R. Nagy, State-of-the-art local correlation methods enable accurate and affordable gold standard quantum chemistry up to a few hundred atoms. *Chem. Sci.* **15**, 14556–14584 (2024).

14. A. Zen, J. G. Brandenburg, J. Klimeš, A. Tkatchenko, D. Alfè, A. Michaelides, Fast and accurate quantum Monte Carlo for molecular crystals. *Proc. Natl. Acad. Sci. U.S.A.* **115**, 1724–1729 (2018).

15. S. Grimme, A. Hansen, J. G. Brandenburg, C. Bannwarth, Dispersion-corrected mean-field electronic structure methods. *Chem. Rev.* **116**, 5105–5154 (2016).

16. P. Xu, M. Alkan, M. S. Gordon, Many-body dispersion. *Chem. Rev.* **120**, 12343–12356 (2020).

17. C. J. Nickerson, K. R. Bryenton, A. J. Price, E. R. Johnson, Comparison of density-functional theory dispersion corrections for the DES15K database. *J. Phys. Chem. A* **127**, 8712–8722 (2023).

18. E. Sim, S. Song, S. Vuckovic, K. Burke, Improving results by improving densities: Density-corrected density functional theory. *J. Am. Chem. Soc.* **144**, 6625–6639 (2022).

19. E. R. Johnson, A. D. Becke, A post-Hartree-Fock model of intermolecular interactions: Inclusion of higher-order corrections. *J. Chem. Phys.* **124**, 174104 (2006).

20. A. D. Becke, E. R. Johnson, Exchange-hole dipole moment and the dispersion interaction revisited. *J. Chem. Phys.* **127**, 154108 (2007).

21. A. Tkatchenko, M. Scheffler, Accurate molecular van der Waals interactions from ground-state electron density and free-atom reference data. *Phys. Rev. Lett.* **102**, 073005 (2009).

22. S. Grimme, J. Antony, S. Ehrlich, H. Krieg, A consistent and accurate ab initio parametrization of density functional dispersion correction (DFT-D) for the 94 elements H-Pu. *J. Chem. Phys.* **132**, 154104 (2010).

23. S. Grimme, S. Ehrlich, L. Goerigk, Effect of the damping function in dispersion corrected density functional theory. *J. Comput. Chem.* **32**, 1456–1465 (2011).

24. J. Klimeš, D. R. Bowler, A. Michaelides, Chemical accuracy for the van der Waals density functional. *J. Phys. Condens. Matter* **22**, 022201 (2010).

25. O. A. Vydrov, T. Van Voorhis, Nonlocal van der Waals density functional: The simpler the better. *J. Chem. Phys.* **133**, 244103 (2010).

26. A. Tkatchenko, R. A. DiStasio Jr., R. Car, M. Scheffler, Accurate and efficient method for many-body van der Waals interactions. *Phys. Rev. Lett.* **108**, 236402 (2012).

27. A. Ambrosetti, A. M. Reilly, R. A. DiStasio, A. Tkatchenko, Long-range correlation energy calculated from coupled atomic response functions. *J. Chem. Phys.* **140**, 18A508 (2014).

28. S. Spicher, E. Caldeweyher, A. Hansen, S. Grimme, Benchmarking London dispersion corrected density functional theory for noncovalent ion–π interactions. *Phys. Chem. Chem. Phys.* **23**, 11635–11648 (2021).

29. V. Wineman-Fisher, Y. Al-Hamdani, P. R. Nagy, A. Tkatchenko, S. Varma, Improved description of ligand polarization enhances transferability of ion–ligand interactions. *J. Chem. Phys.* **153**, 094115 (2020).

30. I. Sandler, S. Sharma, B. Chan, J. Ho, Accurate quantum chemical prediction of gas-phase anion binding affinities and their structure-binding relationships. *J. Phys. Chem. A* **125**, 9838–9851 (2021).

31. A. G. Donchev, A. G. Taube, E. Decolvenaere, C. Hargus, R. T. McGibbon, K. H. Law, B. A. Gregersen, J. L. Li, K. Palmo, K. Siva, M. Bergdorf, J. L. Klepeis, D. E. Shaw, Quantum chemical benchmark databases of gold-standard dimer interaction energies. *Sci. Data* **8**, 55 (2021).

32. E. Caldeweyher, S. Ehlert, A. Hansen, H. Neugebauer, S. Spicher, C. Bannwarth, S. Grimme, A generally applicable atomic-charge dependent London dispersion correction. *J. Chem. Phys.* **150**, 154122 (2019).

33. E. Caldeweyher, J.-M. Mewes, S. Ehlert, S. Grimme, Extension and evaluation of the D4 London-dispersion model for periodic systems. *Phys. Chem. Chem. Phys.* **22**, 8499–8512 (2020).

34. A. Kleshchonok, A. Tkatchenko, Tailoring van der Waals dispersion interactions with external electric charges. *Nat. Commun.* **9**, 3017 (2018).

35. K. R. Bryenton, A. A. Adeleke, S. G. Dale, E. R. Johnson, Delocalization error: The greatest outstanding challenge in density-functional theory. *Wiley Interdiscip. Rev. Comput. Mol. Sci.* **13**, e1631 (2023).

36. E. Palos, A. Caruso, F. Paesani, Consistent density functional theory-based description of ion hydration through density-corrected many-body representations. *J. Chem. Phys.* **159**, 181101 (2023).

37. K. Pernal, R. Podeszwa, K. Patkowski, K. Szalewicz, Dispersionless density functional theory. *Phys. Rev. Lett.* **103**, 263201 (2009).

38. A. J. Price, K. R. Bryenton, E. R. Johnson, Requirements for an accurate dispersion-corrected density functional. *J. Chem. Phys.* **154**, 230902 (2021).

39. O. T. Unke, M. Stöhr, S. Ganscha, T. Unterthiner, H. Maennel, S. Kashubin, D. Ahlin, M. Gastegger, L. Medrano Sandonas, J. T. Berryman, A. Tkatchenko, K. R. Müller, Biomolecular dynamics with machine-learned quantum-mechanical force fields trained on diverse chemical fragments. *Sci. Adv.* **10**, eadn4397 (2024).

40. O. T. Unke, S. Chmiela, H. E. Sauceda, M. Gastegger, I. Poltavsky, K. T. Schütt, A. Tkatchenko, K. R. Müller, Machine learning force fields. *Chem. Rev.* **121**, 10142–10186 (2021).

41. P. Montero de Hijes, C. Dellago, R. Jinnouchi, G. Kresse, Density isobar of water and melting temperature of ice: Assessing common density functionals. *J. Chem. Phys.* **161**, 131102 (2024).

42. E. Palos, E. Lambros, S. Swee, J. Hu, S. Dasgupta, F. Paesani, Assessing the interplay between functional-driven and density-driven errors in DFT models of water. *J. Chem. Theory Comput.* **18**, 3410–3426 (2022).

43. T. W. Ko, J. A. Finkler, S. Goedecker, J. Behler, A fourth-generation high-dimensional neural network potential with accurate electrostatics including non-local charge transfer. *Nat. Commun.* **12**, 398 (2021).

44. J. W. Furness, A. D. Kaplan, J. Ning, J. P. Perdew, J. Sun, Accurate and numerically efficient r^2SCAN meta-generalized gradient approximation. *J. Phys. Chem. Lett.* **11**, 8208–8215 (2020).

45. J. Sun, A. Ruzsinszky, J. P. Perdew, Strongly constrained and appropriately normed semilocal density functional. *Phys. Rev. Lett.* **115**, 036402 (2015).

46. P. M. Gill, B. G. Johnson, J. A. Pople, M. J. Frisch, The performance of the Becke–Lee–Yang–Parr (B–LYP) density functional theory with various basis sets. *Chem. Phys. Lett.* **197**, 499–505 (1992).

47. E. Sim, S. Song, K. Burke, Quantifying density errors in DFT. *J. Phys. Chem. Lett.* **9**, 6385–6392 (2018).

48. M.-C. Kim, E. Sim, K. Burke, Understanding and reducing errors in density functional calculations. *Phys. Rev. Lett.* **111**, 073003 (2013).

49. S. Vuckovic, S. Song, J. Kozlowski, E. Sim, K. Burke, Density functional analysis: The theory of density-corrected DFT. *J. Chem. Theory Comput.* **15**, 6636–6646 (2019).

50. S. Song, S. Vuckovic, E. Sim, K. Burke, Density-corrected DFT explained: Questions and answers. *J. Chem. Theory Comput.* **18**, 817–827 (2022).

51. S. Song, S. Vuckovic, E. Sim, K. Burke, Density sensitivity of empirical functionals. *J. Phys. Chem. Lett.* **12**, 800–807 (2021).

52. S. Song, S. Vuckovic, Y. Kim, H. Yu, E. Sim, K. Burke, Extending density functional theory with near chemical accuracy beyond pure water. *Nat. Commun.* **14**, 799 (2023).

53. M. Lee, B. Kim, M. Sim, M. Sogal, Y. Kim, H. Yu, K. Burke, E. Sim, Correcting dispersion corrections with density-corrected DFT. *J. Chem. Theory Comput.* **20**, 7155–7167 (2024).

54. T. Gould, B. Chan, S. G. Dale, S. Vuckovic, Identifying and embedding transferability in data-driven representations of chemical space. *Chem. Sci.* **15**, 11122–11133 (2024).

55. J. Hoja, L. Medrano Sandonas, B. G. Ernst, A. Vazquez-Mayagoitia, R. A. DiStasio, A. Tkatchenko, QM7-X, a comprehensive dataset of quantum-mechanical properties spanning the chemical space of small organic molecules. *Sci. Data* **8**, 43 (2021).

56. L. Medrano Sandonas, D. van Rompaey, A. Fallani, M. Hilfiker, D. Hahn, L. Perez-Benito, J. Verhoeven, G. Tresadern, J. Kurt Wegner, H. Ceulemans, A. Tkatchenko, Dataset for quantum-mechanical exploration of conformers and solvent effects in large drug-like molecules. *Sci. Data* **11**, 742 (2024).

57. A. Bauza, I. Alkorta, A. Frontera, J. Elguero, On the reliability of pure and hybrid DFT methods for the evaluation of halogen, chalcogen, and pnicogen bonds involving anionic and neutral electron donors. *J. Chem. Theory Comput.* **9**, 5201–5210 (2013).

58. L. Goerigk, A. Hansen, C. Bauer, S. Ehrlich, A. Najibi, S. Grimme, A look at the density functional theory zoo with the advanced GMTKN55 database for general main group thermochemistry, kinetics and noncovalent interactions. *Phys. Chem. Chem. Phys.* **19**, 32184–32215 (2017).

59. N. H. Williams, Magnesium ion catalyzed ATP hydrolysis. *J. Am. Chem. Soc.* **122**, 12023–12024 (2000).

60. H. Noguchi, T. Ikegami, A. Nagadoi, Y. O. Kamatari, S. Y. Park, J. R. H. Tame, S. Unzai, The structure and conformational switching of Rap1B. *Biochem. Biophys. Res. Commun.* **462**, 46–51 (2015).

61. J. Bröker, A. G. Waterson, T. R. Hodges, J. R. Abbott, A. Arnold, J. Böttcher, N. Braun, J. Cui, J. E. Fuchs, T. Gerstberger, S. Gogg, S. Hanner, L. Herdeis, L. W. Howell, A. Mantoulidis, M. Mayer, J. Phan, F. Rocchetti, K. Sankar, D. Sarkar, O. Schaaf, J. L. Sensintaffar, Q. Sun, T. Wunberg, S. W. Fesik, Discovery of BI-2493, a pan-KRAS inhibitor showing in vivo efficacy. *J. Med. Chem.* **68**, 15649–15668 (2025).

62. J. H. Laity, B. M. Lee, P. E. Wright, Zinc finger proteins: New insights into structural and functional diversity. *Curr. Opin. Struct. Biol.* **11**, 39–46 (2001).

63. Z. Yu, P. Li, K. M. Merz, Extended zinc AMBER force field (EZAFF). *J. Chem. Theory Comput.* **14**, 242–254 (2017).

64. I. Dokmanić, M. Šikić, S. Tomić, Metals in proteins: Correlation between the metal-ion type, coordination number and the amino-acid residues involved in the coordination. *Acta Crystallogr. D.* **64**, 257–263 (2008).

65. V. Putignano, A. Rosato, L. Banci, C. Andreini, MetalPDB in 2018: A database of metal sites in biological macromolecular structures. *Nucleic Acids Res.* **46**, D459–D464 (2018).

66. T. R. Soderling, J. T. Stull, Structure and regulation of calcium/calmodulin-dependent protein kinases. *Chem. Rev.* **101**, 2341–2352 (2001).

67. D. L. Scott, S. P. White, J. L. Browning, J. J. Rosa, M. H. Gelb, P. B. Sigler, Structures of free and inhibited human secretory phospholipase A2 from inflammatory exudate. *Science* **254**, 1007–1010 (1991).

68. A. Teplyakov, G. Obmolova, P. P. Khil, A. J. Howard, R. D. Camerini-Otero, G. L. Gilliland, Crystal structure of the *Escherichia coli* YcdX protein reveals a trinuclear zinc active site. *Proteins* **51**, 315–318 (2003).

69. A. Echalier, C. F. Goodhew, G. W. Pettigrew, V. Fülöp, Activation and catalysis of the di-heme cytochrome c peroxidase from *Paracoccus pantotrophus*. *Structure* **14**, 107–117 (2006).

70. S. O. Dahms, T. Haider, G. Klebe, T. Steinmetzer, H. Brandstetter, OFF-state-specific inhibition of the proprotein convertase furin. *ACS Chem. Biol.* **16**, 1692–1700 (2021).

71. S. K. Singh, A. Yamashita, E. Gouaux, Antidepressant binding site in a bacterial homologue of neurotransmitter transporters. *Nature* **448**, 952–956 (2007).

72. H. Matsumura, T. Yamamoto, T. C. Leow, T. Mori, A. B. Salleh, M. Basri, T. Inoue, Y. Kai, R. N. Z. R. A. Rahman, Novel cation-π interaction revealed by crystal structure of thermoalkalophilic lipase. *Proteins* **70**, 592–598 (2008).

73. P. R. Nagy, M. Kállay, Optimization of the linear-scaling local natural orbital CCSD(T) method: Redundancy-free triples correction using Laplace transform. *J. Chem. Phys.* **146**, 214106 (2017).

74. P. R. Nagy, G. Samu, M. Kállay, Optimization of the linear-scaling local natural orbital CCSD(T) method: Improved algorithm and benchmark applications. *J. Chem. Theory Comput.* **14**, 4193–4215 (2018).

75. A. Mirtschink, C. J. Umrigar, J. D. Morgan III, P. Gori-Giorgi, Energy density functionals from the strong-coupling limit applied to the anions of the He isoelectronic series. *J. Chem. Phys.* **140**, 18A532 (2014).

76. J. Hermann, M. Stöhr, S. Góger, S. Chaudhuri, B. Aradi, R. J. Maurer, A. Tkatchenko, libMBD: A general-purpose package for scalable quantum many-body dispersion calculations. *J. Chem. Phys.* **159**, 174802 (2023).

77. V. Wineman-Fisher, J. M. Delgado, P. R. Nagy, E. Jakobsson, S. A. Pandit, S. Varma, Transferable interactions of Li^{+} and Mg^{2+} ions in polarizable models. *J. Chem. Phys.* **153**, 104113 (2020).

78. J. Chen, B. Chan, Y. Shao, J. Ho, How accurate are approximate quantum chemical methods at modelling solute-solvent interactions in solvated clusters? *Phys. Chem. Chem. Phys.* **22**, 3855–3866 (2020).

79. S. A. Slattery, J. C. Yon, E. F. Valeev, Revisiting artifacts of Kohn–Sham density functionals for biosimulation. *J. Chem. Theory Comput.* **20**, 6652–6660 (2024).

80. S. Nam, S. Song, E. Sim, K. Burke, Measuring density-driven errors using Kohn–Sham inversion. *J. Chem. Theory Comput.* **16**, 5014–5023 (2020).

81. A. D. Kaplan, C. Shahi, P. Bhetwal, R. K. Sah, J. P. Perdew, Understanding density-driven errors for reaction barrier heights. *J. Chem. Theory Comput.* **19**, 532–543 (2023).

82. B. Kanungo, A. D. Kaplan, C. Shahi, V. Gavini, J. P. Perdew, Unconventional error cancellation explains the success of Hartree–Fock density functional theory for barrier heights. *J. Phys. Chem. Lett.* **15**, 323–328 (2024).

83. H. Bahmann, Y. Zhou, M. Ernzerhof, The shell model for the exchange-correlation hole in the strong-correlation limit. *J. Chem. Phys.* **145**, 124104 (2016).

84. S. Vuckovic, P. Gori-Giorgi, Simple fully nonlocal density functionals for electronic repulsion energy. *J. Phys. Chem. Lett.* **8**, 2799–2805 (2017).

85. S. Vuckovic, H. Bahmann, Nonlocal functionals inspired by the strongly interacting limit of DFT: Exact constraints and implementation. *J. Chem. Theory Comput.* **19**, 6172–6184 (2023).

86. T. Froitzheim, M. Müller, A. Hansen, S. Grimme, The bond capacity electronegativity equilibration charge model (EEQ_{BC}) for the elements $Z = 1$–103. *J. Chem. Phys.* **162**, 214109 (2025).

87. Y. Zhao, D. G. Truhlar, The M06 suite of density functionals for main group thermochemistry, thermochemical kinetics, noncovalent interactions, excited states, and transition elements: Two new functionals and systematic testing of four M06-class functionals and 12 other functionals. *Theor. Chem. Acc.* **120**, 215–241 (2008).

88. J. Tao, J. P. Perdew, V. N. Staroverov, G. E. Scuseria, Climbing the density functional ladder: Nonempirical meta–generalized gradient approximation designed for molecules and solids. *Phys. Rev. Lett.* **91**, 146401 (2003).

89. M. Bursch, H. Neugebauer, S. Ehlert, S. Grimme, Dispersion corrected r^2SCAN based global hybrid functionals: r^2SCANh, r^2SCAN0, and r^2SCAN50. *J. Chem. Phys.* **156**, 134105 (2022).

90. N. Mardirossian, M. Head-Gordon, ωB97M-V: A combinatorially optimized, range-separated hybrid, meta-GGA density functional with VV10 nonlocal correlation. *J. Chem. Phys.* **144**, 214110 (2016).

91. G. Santra, N. Sylvetsky, J. M. Martin, Minimally empirical double-hybrid functionals trained against the GMTKN55 database: revDSD-PBEP86-D4, revDOD-PBE-D4, and DOD-SCAN-D4. *J. Phys. Chem. A* **123**, 5129–5143 (2019).

92. R. Recabarren, K. Zinovjev, I. Tuñón, J. Alzate-Morales, How a second Mg^{2+} ion affects the phosphoryl-transfer mechanism in a protein kinase: A computational study. *ACS Catal.* **11**, 169–183 (2020).

93. K. Nam, A. R. A. Thodika, S. Tischlik, C. Phoeurk, T. M. Nagy, L. Schierholz, J. Ådén, P. Rogne, M. Drescher, A. E. Sauer-Eriksson, M. Wolf-Watz, Magnesium induced structural reorganization in the active site of adenylate kinase. *Sci. Adv.* **10**, eado5504 (2024).

94. U. Kökçam-Demir, A. Goldman, L. Esrafili, M. Gharib, A. Morsali, O. Weingart, C. Janiak, Coordinatively unsaturated metal sites (open metal sites) in metal-organic frameworks: Design and applications. *Chem. Soc. Rev.* **49**, 2751–2798 (2020).

95. A. L. Dzubak, L. C. Lin, J. Kim, J. A. Swisher, R. Poloni, S. N. Maximoff, B. Smit, L. Gagliardi, Ab initio carbon capture in open-site metal-organic frameworks. *Nat. Chem.* **4**, 810–816 (2012).

96. R. Goeminne, L. Vanduyfhuys, V. Van Speybroeck, T. Verstraelen, DFT-quality adsorption simulations in metal–organic frameworks enabled by machine learning potentials. *J. Chem. Theory Comput.* **19**, 6313–6325 (2023).

97. B. Rana, M. P. Coons, J. M. Herbert, Detection and correction of delocalization errors for electron and hole polarons using density-corrected DFT. *J. Phys. Chem. Lett.* **13**, 5275–5284 (2022).

98. F. Belleflamme, J. Hutter, Radicals in aqueous solution: Assessment of density-corrected SCAN functional. *Phys. Chem. Chem. Phys.* **25**, 20817–20836 (2023).

99. Q. Sun, X. Zhang, S. Banerjee, P. Bao, M. Barbry, N. S. Blunt, N. A. Bogdanov, G. H. Booth, J. Chen, Z. H. Cui, J. J. Eriksen, Y. Gao, S. Guo, J. Hermann, M. R. Hermes, K. Koh, P. Koval, S. Lehtola, Z. Li, J. Liu, N. Mardirossian, J. D. McClain, M. Motta, B. Mussard, H. Q. Pham, A. Pulkin, W. Purwanto, P. J. Robinson, E. Ronca, E. R. Sayfutyarova, M. Scheurer, H. F. Schurkus, J. E. T. Smith, C. Sun, S. N. Sun, S. Upadhyay, L. K. Wagner, X. Wang, A. White, J. D. Whitfield, M. J. Williamson, S. Wouters, J. Yang, J. M. Yu, T. Zhu, T. C. Berkelbach, S. Sharma, A. Y. Sokolov, G. K. L. Chan, Recent developments in the PySCF program package. *J. Chem. Phys.* **153**, 024109 (2020).

100. F. Neese, Software update: The ORCA program system—Version 5.0. *Wiley Interdiscip. Rev. Comput. Mol. Sci.* **12**, e1606 (2022).

101. F. Weigend, R. Ahlrichs, Balanced basis sets of split valence, triple zeta valence and quadruple zeta valence quality for H to Rn: Design and assessment of accuracy. *Phys. Chem. Chem. Phys.* **7**, 3297–3305 (2005).

102. F. Weigend, Hartree–Fock exchange fitting basis sets for H to Rn. *J. Comput. Chem.* **29**, 167–175 (2008).

103. J. Hermann, R. A. DiStasio Jr., A. Tkatchenko, First-principles models for van der Waals interactions in molecules and materials: Concepts, theory, and applications. *Chem. Rev.* **117**, 4714–4758 (2017).

104. B. Brauer, M. K. Kesharwani, S. Kozuch, J. M. Martin, The S66x8 benchmark for noncovalent interactions revisited: Explicitly correlated ab initio methods and density functional theory. *Phys. Chem. Chem. Phys.* **18**, 20905–20925 (2016).

105. P. Agrawal, S. Patiyal, R. Kumar, V. Kumar, H. Singh, P. K. Raghav, G. P. S. Raghava, ccPDB 2.0: An updated version of datasets created and compiled from Protein Data Bank. *Database* **2019**, bay142 (2019).

106. J. A. Delgado, V. Wineman-Fisher, S. Pandit, S. Varma, Inclusion of high-field target data in AMOEBA's calibration improves predictions of protein-ion interactions. *J. Chem. Inf. Model.* **62**, 4713–4726 (2022).

107. S. Jo, T. Kim, V. G. Iyer, W. Im, CHARMM-GUI: A web-based graphical user interface for CHARMM. *J. Comput. Chem.* **29**, 1859–1865 (2008).

108. A. T. Brünger, M. Karplus, Polar hydrogen positions in proteins: Empirical energy placement and neutron diffraction comparison. *Proteins* **4**, 148–156 (1988).

109. M. Kállay, P. R. Nagy, D. Mester, Z. Rolik, G. Samu, J. Csontos, J. Csóka, P. B. Szabó, L. Gyevi-Nagy, B. Hégely, I. Ladjánszki, L. Szegedy, B. Ladóczki, K. Petrov, M. Farkas, P. D. Mezei, Á. Ganyecz, The MRCC program system: Accurate quantum chemistry from water to proteins. *J. Chem. Phys.* **152**, 074107 (2020).

110. D. Mester, P. R. Nagy, J. Csóka, L. Gyevi-Nagy, P. B. Szabó, R. A. Horváth, K. Petrov, B. Hégely, B. Ladóczki, G. Samu, B. D. Lőrincz, M. Kállay, Overview of developments in the MRCC program system. *J. Phys. Chem. A* **129**, 2086–2107 (2025).

111. M. Kállay, P. R. Nagy, D. Mester, L. Gyevi-Nagy, J. Csóka, P. B. Szabó, Z. Rolik, G. Samu, J. Csontos, B. Hégely, MRCC, a quantum chemical program suite. www.mrcc.hu/ [accessed 1 August 2023].

112. S. F. Boys, F. Bernardi, The calculation of small molecular interactions by the differences of separate total energies. Some procedures with reduced errors. *Mol. Phys.* **19**, 553–566 (1970).

113. D. Mester, P. R. Nagy, M. Kállay, Basis-set limit CCSD(T) energies for large molecules with local natural orbitals and reduced-scaling basis-set corrections. *J. Chem. Theory Comput.* **20**, 7453–7468 (2024).

114. T. H. Dunning Jr., K. A. Peterson, A. K. Wilson, Gaussian basis sets for use in correlated molecular calculations. X. The atoms aluminum through argon revisited. *J. Chem. Phys.* **114**, 9244–9253 (2001).

115. T. Helgaker, W. Klopper, H. Koch, J. Noga, Basis-set convergence of correlated calculations on water. *J. Chem. Phys.* **106**, 9639–9646 (1997).

116. P. R. Nagy, G. Samu, M. Kállay, An integral-direct linear-scaling second-order Mø ller–Plesset approach. *J. Chem. Theory Comput.* **12**, 4897–4914 (2016).

117. B. P. Prascher, D. E. Woon, K. A. Peterson, T. H. Dunning, A. K. Wilson, Gaussian basis sets for use in correlated molecular calculations. VII. Valence, core-valence, and scalar relativistic basis sets for Li, Be, Na, and Mg. *Theor. Chem. Acc.* **128**, 69–82 (2011).

118. J. G. Hill, K. A. Peterson, Gaussian basis sets for use in correlated molecular calculations. XI. Pseudopotential-based and all-electron relativistic basis sets for alkali metal (K-Fr) and alkaline earth (Ca-Ra) elements. *J. Chem. Phys.* **147**, 244106 (2017).

119. D. Figgen, G. Rauhut, M. Dolg, H. Stoll, Energy-consistent pseudopotentials for group 11 and 12 atoms: Adjustment to multi-configuration Dirac–Hartree–Fock data. *Chem. Phys.* **311**, 227–244 (2005).

120. A. Karton, J. M. L. Martin, Comment on: "Estimating the Hartree–Fock limit from finite basis set calculations" [Jensen F (2005) Theor Chem Acc 113:267]. *Theor. Chem. Acc.* **115**, 330–333 (2006).

121. E. Giner, B. Pradines, A. Ferté, R. Assaraf, A. Savin, J. Toulouse, Curing basis-set convergence of wave-function theory using density-functional theory: A systematically improvable approach. *J. Chem. Phys.* **149**, 194301 (2018).

122. F. Weigend, A. Köhn, C. Hättig, Efficient use of the correlation consistent basis sets in resolution of the identity MP2 calculations. *J. Chem. Phys.* **116**, 3175–3183 (2002).

123. C. Hättig, Optimization of auxiliary basis sets for RI-MP2 and RI-CC2 calculations: Core-valence and quintuple-ζ basis sets for H to Ar and QZVPP basis sets for Li to Kr. *Phys. Chem. Chem. Phys.* **7**, 59–66 (2005).

124. J. G. Hill, Auxiliary basis sets for density-fitting second-order Mø ller–Plesset perturbation theory: Weighted core-valence correlation consistent basis sets for the 4d elements Y-Pd. *J. Comput. Chem.* **34**, 2168–2177 (2013).
